# Development of a Mobile Intervention for Procrastination Augmented With a Semigenerative Chatbot for University Students: Pilot Randomized Controlled Trial

**DOI:** 10.2196/53133

**Published:** 2025-04-10

**Authors:** Seonmi Lee, Jaehyun Jeong, Myungsung Kim, Sangil Lee, Sung-Phil Kim, Dooyoung Jung

**Affiliations:** 1 Graduate School of Health Science and Technology Ulsan National Institute of Science and Technology Ulsan Republic of Korea; 2 Department of Biomedical Engineering Ulsan National Institute of Science and Technology Ulsan Republic of Korea; 3 LVIS Korea Daegu Republic of Korea; 4 School of Liberal Arts Ulsan National Institute of Science and Technology Ulsan Republic of Korea

**Keywords:** procrastination, chatbot, generative model, semigenerative model, time management, cognitive behavioral therapy, psychological assessment, intervention engagement, emotional support, user experience, mobile intervention, artificial intelligence, AI

## Abstract

**Background:**

Procrastination negatively affects university students’ academics and mental health. Traditional time management apps lack therapeutic strategies like cognitive behavioral therapy to address procrastination’s psychological aspects. Therefore, we developed and integrated a semigenerative chatbot named Moa into a to-do app.

**Objective:**

We intended to determine the benefits of the Moa-integrated to-do app over the app without Moa by verifying behavioral and cognitive changes, analyzing the influence of engagement patterns on the changes, and exploring the user experience.

**Methods:**

The developed chatbot Moa guided users over 30 days in terms of self-observation, strategy establishment, and reflection. The architecture comprised response-generating and procrastination factor–detection algorithms. A pilot randomized controlled trial was conducted with 85 participants (n=37, 44% female; n=48, 56% male) from a university in South Korea. The control group used a to-do app without Moa, whereas the treatment group used a fully automated Moa-integrated app. The Irrational Procrastination Scale, Pure Procrastination Scale, Time Management Behavior Scale, and the Perceived Stress Scale were examined using linear mixed models with repeated measurements obtained before (T0) and after (T1) 1-month use and after 2-month use (T2) to assess the changes in irrational procrastination, pure procrastination, time management and behavior, academic self-regulation, and stress. Intervention engagement, divided into “high,” “middle” and “low” clusters, was quantified using app access and use of the to-do list and grouped using k-means clustering. In addition, changes in the psychological scale scores between the control and treatment groups were analyzed within each cluster. User experience was quantified based on the usability, feasibility, and acceptability of and satisfaction with the app, whereas thematic analysis explored the users’ subjective responses to app use.

**Results:**

In total, 75 participants completed the study. The interaction of time × procrastination was significant during the required use period (*P*=.01). The post hoc test indicated a significant improvement from T0 to T1 in the Time Management Behavior Scale and Perceived Stress Scale scores only in the treatment group (*P*<.001 and *P*=.009). The changes in Pure Procrastination Scale score after the required use period were significant in all clusters except for the low cluster of the control group. The high cluster in the treatment group exhibited a significant change in the Irrational Procrastination Scale after Bonferroni correction (*P*=.046). Usability was determined to be good in the treatment group (mean score 72.8, SD 16.0), and acceptability was higher than in the control group (*P*=.03). Evaluation of user experience indicated that only the participants in the treatment group achieved self-reflection and experienced an alliance with the app.

**Conclusions:**

The chatbot-integrated app demonstrated greater efficacy in influencing user behavior providing psychological support. It will serve as a valuable tool for managing procrastination and stress together.

**Trial Registration:**

Clinical Research Information Service (CRIS) KCT0009056; https://tinyurl.com/yc84tedk

## Introduction

### Background

In psychology, procrastination refers to delaying an intended course of action despite expecting that this will lead to a worse outcome [1]. Rothblum et al [2] reported that fear of failure and task aversiveness are the primary causes of procrastination, indicating that students who feel overwhelmed by tasks delay the action further. Prolonged procrastination leads to poor academic performance, which impacts the careers of university students, elevates stress, and forms a negative cycle that postpones work [3,4].

Time management apps such as to-do apps are convenient for controlling this delaying behavior for better performance. Time management involves “behaviors that aim at achieving an effective use of time while performing certain goal-directed activities” [5]. Methods for implementing time management strategies such as planning and monitoring have been adapted to mobile apps to enable users to manage their tasks efficiently and effectively [6]. For instance, certain apps assist in subdividing tasks into smaller goals or performing self-assessment by maintaining daily journals [7-9].

However, existing time management apps face challenges in fundamentally resolving the issue of procrastination. They often do not manage various aspects of procrastination, including self-esteem, motivation, and mood regulation [10]. Procrastination is not solely a matter of time management but is closely associated with psychological problems such as low life satisfaction and poor mental health [11,12]. Recognizing procrastination as a psychological issue, psychologists have adapted cognitive behavioral therapy (CBT) programs to help students identify irrational thought processes, which in turn can motivate them to change their behavior [13]. Numerous studies have validated the effectiveness of CBT in treating procrastination [14-16].

However, engaging in offline counseling is often challenging for students due to difficulties in accessing mental health care services compounded by time constraints and a lack of familiarity with the therapeutic approaches used to address procrastination [17]. Consequently, they may prefer using time management apps for self-management such as Google Keep [18] or TickTick [19] owing to their convenience, time-saving features, and effectiveness in task management. Typically, these apps do not contain sufficient therapeutic content to raise awareness of irrational behavioral patterns, provide motivation, or assist in behavior change.

In this study, we developed and integrated a chatbot into a time management app to provide sufficient therapeutic content while ensuring convenience. Chatbots are widely used to support task performance in various fields because they efficiently deliver information by interacting with users in natural languages [20]. In addition, chatbots enable emotional interaction in conversations and form alliances that prolong the interest of users in the app [21,22]. Verbal communication, which is the primary feature of chatbots, is the basis of psychological therapy [23]. Both clients and counselors can share their thoughts and feelings verbally, whereas the counselors can lead conversations with adequate questions and responses. These strengths have increased the efforts to use chatbots for psychological assistance in the mental health care field [21,22].

Most health care chatbots are rule based and retrieval based, meaning that they do not use generative models such as GPT-3.5 and LaMDA [24,25]. Rule-based chatbots offer predetermined options for users, ensuring cost-effective information delivery yet limiting conversational flexibility [20]. Retrieval-based chatbots determine responses based on an artificial intelligence model to classify intent, enabling natural language interactions for goal-oriented conversations, such as in CBT [26,27]. Despite their advantages, these chatbots are confined to preexisting responses, hindering various reactions, resulting in limited empathetic conversations, and restricting the formation of alliances from interactions. Generative models address these limitations by providing relevant responses and empowering users to control conversation contents [28,29]. However, relying solely on generative models for therapeutic purposes is challenging because they cannot consistently maintain context and may produce inaccurate outputs, particularly in sensitive discussions related to severe mental health problems [30]. Therefore, a predetermined framework is necessary in the psychological field to avoid distraction from the conversation topic and minimize detrimental effects caused by erroneous responses when using the generative model.

### Objectives

In this study, we developed a semigenerative chatbot named Moa to facilitate natural conversations within a predetermined scenario. This was integrated into a to-do app to mediate procrastination using a psychological approach. The architecture of Moa included a generative model to facilitate flexible responses and was tailored to user responses. Moa was interlocked with a to-do app that primarily included time management skills, planning, and monitoring and was endowed with the functions of listing tasks to be executed and visualizing achievement through a calendar [5]. After integrating Moa into the to-do app, we tested whether the developed chatbot could provide additional benefits over an app that only supports time management without a chatbot. To this end, we conducted a randomized controlled trial (RCT) setting the intervention for the control group as the to-do app without Moa and that for the treatment group as the Moa-integrated to-do app. In addition to assessing the impact of Moa based on differences in survey scores between the control and treatment groups, we examined the changes in scores by clustering the intervention engagement patterns. This analysis acknowledged that the effectiveness of an intervention can significantly vary depending on user engagement [31]. In addition, both quantitative and qualitative analyses of the user experience were conducted to identify the specific elements influenced by Moa. In summary, our study aimed to determine the additional benefits of using the to-do app with Moa in comparison with using the app without Moa. The primary objectives of this study can be summarized as follows: (1) to validate changes in behavior related to procrastination and psychological aspects such as emotions and stress through self-reported survey scores, (2) to analyze the influence of intervention engagement on the improvement in users’ behavior and cognition, and (3) to quantitatively and qualitatively explore the user experience.

## Methods

### Development of the Intervention

#### Overview

**Figure 1:**
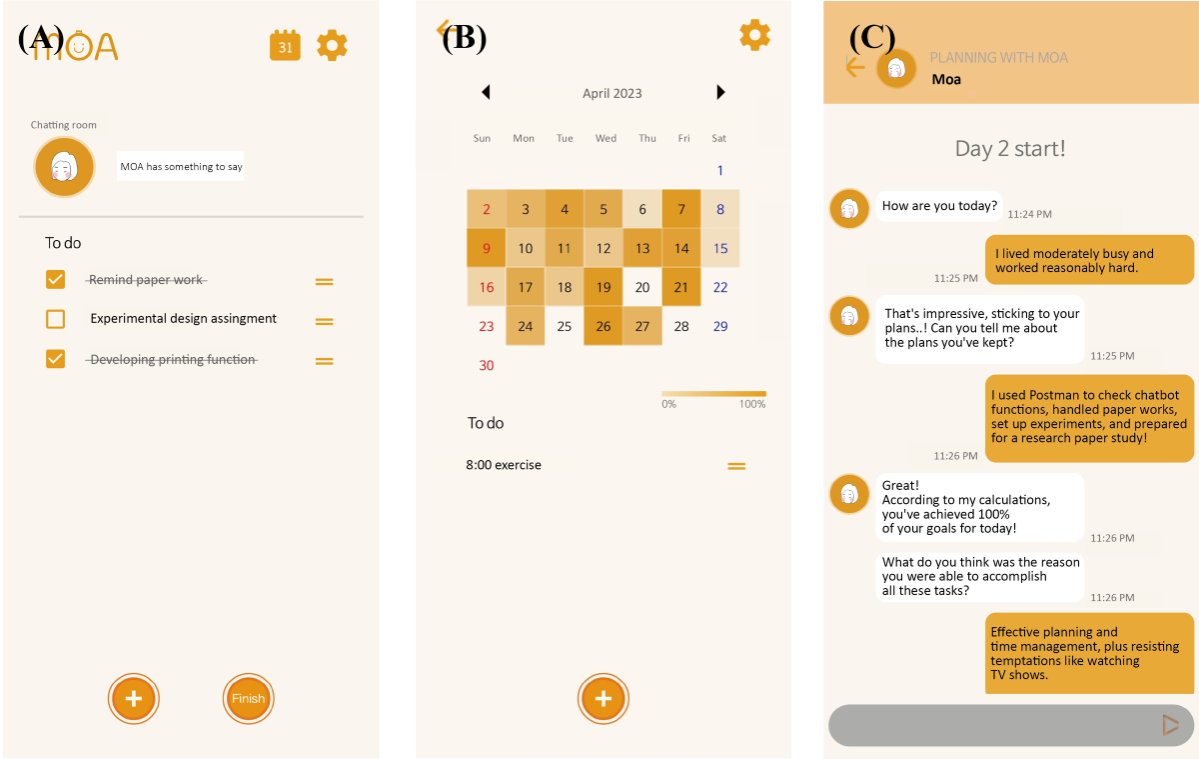
Screenshots of the time management app we used for the treatment group: (A) main screen with to-do list; (B) calendar screen visualizing success rate; (C) chatting room screen for conversation with the chatbot Moa. The content that was originally in Korean has been translated into English.

The intervention for the control group involved performing to-do app functions, and the Moa chatbot was added only for the treatment group. A typical to-do app pushes alarms to write plans in the morning if users do not note any lists for the day. Users tap the Finish button after checking the tasks completed on the list. Subsequently, the success rate, which denotes the ratio of the number of checked lists to the total number of lists, is automatically calculated, and the saturation of the calendar differs from the success rate. For the treatment group, the chatbot Moa initiates a conversation based on the success rate. Figures 1A and 1B depict the overall user interface (UI) of the to-do app.

Moa is a semigenerative chatbot interlocked with the to-do app to facilitate conversations tailored according to the users’ delaying behavior. Moa aims to foster users’ awareness of behavioral patterns and mediate procrastination. To realize the intervention for the treatment group, the function of Moa was interlocked as depicted in Figure 1C.

Moa includes generative models in a predetermined scenario based on the CBT process to encourage active user engagement in conversations while maintaining consistency to guide behavior change; therefore, it is referred to as a semigenerative chatbot. Typically, CBT chatbots for mental health are rule-based systems driven by knowledge-based communication [30]. Generative models of mental health–related chatbots are infrequent because of the potential for erroneous responses, which could lead to major issues such as inadvertent promotion of self-harm or suicide [20]. To minimize the damage from incorrect responses, we restricted the topic of Moa to problems related to procrastination rather than severe mental illnesses. In addition, we incorporated predetermined questionnaires and established a scenario to maintain context within a conversation, ensuring that, even if the chatbot responds awkwardly, the conversation stays on track and avoids harmful directions. These questionnaires were a part of the therapeutic process and provided knowledge pertinent to time management. Furthermore, we collected our own data, and the authors reviewed them to remove harmful utterances from the training dataset used for generative pretrained transformer development.

The overall Moa chatbot scenario comprises the process of procrastination counseling in academic settings that observes maladaptive delaying behavior, searching for its causes and modifying behavior and cognition [32]. In addition, procrastination is highly associated with a lack of self-determined motivation, and most people with high procrastination levels struggle to establish reasonable goals to initiate the tasks [33]. To encourage people to set achievable goals, we adapted the early part of the life-crafting process, which involved identifying life values and goals to motivate users to implement actions and plan accordingly [34]. This scenario was thoroughly reviewed by the coauthors, including a psychiatrist and a clinical psychologist.

The Moa chatbot sessions spanned 1 month and featured varied weekly topics. The sessions included the following steps: (1) observing oneself, (2) understanding strengths and weaknesses while working, (3) establishing personalized strategies by reflecting on one’s behavioral patterns, (4) learning effective strategies, and (5) reflecting on personal change. These sessions encouraged users to develop an awareness of their behavioral patterns and pursue changes. The detailed scenario for each session is provided in Multimedia Appendix 1.

**Figure 2:**
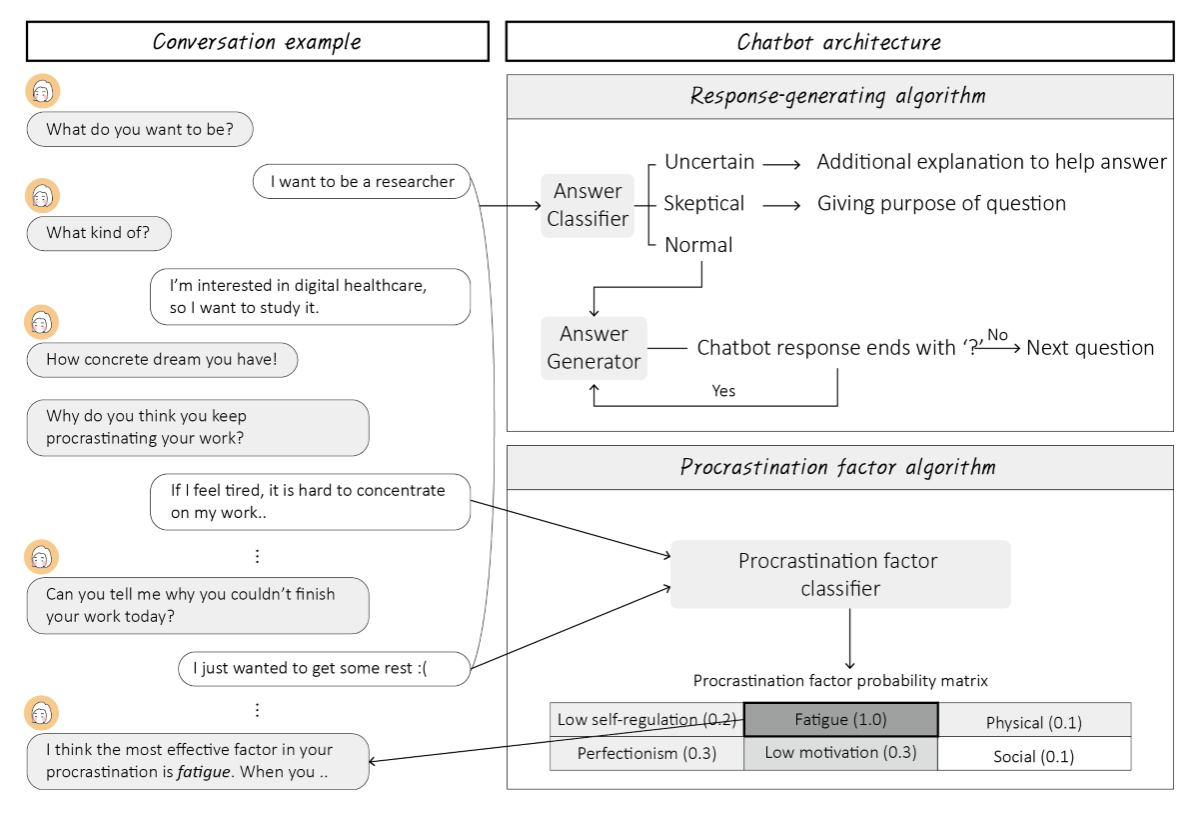
Moa chatbot architecture.

To operate Moa with tailored responses and advice, we designed a chatbot architecture comprising 2 primary algorithms: a response-generating and a procrastination factor algorithm (Figure 2). A survey dataset was collected from researchers, and an open-source dataset was used to train the models embedded in the algorithms. The survey data were obtained via questionnaires designed with open-ended questions. The questionnaires were administered between 2021 and 2022 to 447 undergraduate students at a university in South Korea. Survey data were used after elaboration to train each model. We also used an open-source dataset, namely, the wellness conversation script dataset publicly available from the AI Hub, an open-data web platform [35]. This dataset comprises sentence pairs with problem statements and the corresponding chatbot responses. Sentences labeled as issues that college students commonly face, such as emotional regulation difficulties, worries, self-blame, and interpersonal relationships, were selected; a total of 765 sentence pairs were included.

#### Response-Generating Algorithm

The response-generating algorithm describes how Moa responds to user utterances, as depicted in Figure 2. When the user responds to the question posed by Moa, the answer classifier classifies the user’s response into 1 of 3 categories: “uncertain,” “skeptical,” or “normal.” A response classified as uncertain or skeptical indicates a situation in which the user experiences difficulty in responding. In such cases, the algorithm retrieves sentences from the corpus to help the user reconsider the question. If the user’s response is classified as normal, the answer generator generates a sentence using the response. When an interrogative sentence is generated, the algorithm waits for the subsequent response from the user. Conversely, in the case of a declarative sentence, the algorithm proceeds to the subsequent step in the scenario.

The answer classifier was trained using the Korean Bidirectional Encoder Representations From Transformers [36] with 3356 survey data sentences labeled as uncertain, skeptical, or normal. The test accuracy of the model was 0.86 when the training and test datasets were used in the ratio of 7:3. The answer generator used the Korean generative pretrained transformer 2 [37] released by SK Telecom and fine-tuned it using the data suitable for the scenario. A total of 18,453 sentences from the survey data and the open-source dataset were used for fine-tuning.

#### Procrastination Factor Algorithm

The procrastination factor algorithm was designed to detect the leading causes of procrastination based on user utterances to provide tailored advice. Initially, we conducted a literature review [1,38-46] using the keywords “procrastination,” “risk factors,” and “students” to define the principal aspects of procrastination. The reasons for procrastination from each reference were extracted and categorized within common themes, and 6 factors were identified: physical and emotional stress, social relationships, environmental distractions, perfectionism, low self-regulation, and lack of motivation. The results of the literature review can be found in Multimedia Appendix 2. These factors served as labels for the training datasets.

We trained a multilayered convolutional neural network using 719 sentences from the survey data to develop a procrastination factor classifier. These sentences were divided in the ratio of 7:3 for training and test sets to validate the model. We achieved an accuracy of 51%, with a top-2 accuracy of 81%. The classifier was adopted to answer several questions related to work inefficiency, and the resulting probability matrices were combined to improve the ability to detect procrastination factors. The chatbot provided tailored advice relevant to the factor with the highest score in the final matrix. If the user disagreed with the results inferred from the model, one of the provided advices for the second-highest factor was randomly selected and recommended, and the top 1 and 2 values from the matrix were switched and saved to reflect the genuine information. To generate content for advice, we conducted brief discussions with 4 certified clinical psychologists. These counselors presented advice on the role of Moa in situations in which users could not complete their work owing to each procrastination factor. Refined responses were saved in a database for generating tailored advice.

### Study Design

To validate the effect of the Moa-integrated to-do app, we conducted a pilot study using a split-plot RCT design among university students.

### Ethical Considerations

Facilities Ethics Board approved the experimental procedures of the Ulsan National Institute of Science and Technology (UNISTIRB-22-72-A). Every participant provided their consent via email after they received the explanation of the overall experiment process through a web-based meeting. This study did not collect personally identifiable information from participants, and each individual’s data were deidentified. Participants were compensated based on the duration of their involvement in the experiment, with those who completed the 2-month experiment and submitted the questionnaire receiving approximately US $70.

### Participants

In March 2023, a total of 151 undergraduate students from a Korean university were initially selected to participate in this experiment. We recruited participants through social media and campus posters using the following statement: “Recruitment for user test of an application for effective time management.” Participants were not informed of the specific characteristics of the interventions, which allowed us to blind participants to whether they were in the treatment or control group. Interested individuals voluntarily completed web-based application forms containing recruitment surveys related to demographic information and the Patient Health Questionnaire–9 (PHQ-9), Generalized Anxiety Disorder–7 (GAD-7), Pure Procrastination Scale (PPS), and Irrational Procrastination Scale (IPS) to determine their eligibility for participating in the experiment.

We focused on individuals who had procrastination without other severe mental health conditions such as depression and anxiety. Therefore, individuals scoring <29 on the PPS, the average score in a previous study that assessed PPS scores from Korean university students, were not allowed to participate in the experiment [47]. The presence of other mental health conditions was examined based on the severity indicated by the PHQ-9 and GAD-7, and individuals with scores of >14 on both these scales, indicating a cutoff for a severe level, were excluded to avoid including those with potentially severe mood disorders that required clinical treatment initially [48,49].

Participants who failed to use the to-do list app with the chatbot at least 3 times during a 1-week practice session were also excluded to ensure consistent engagement throughout the experiment.

After the practice session, participants were asked to complete a baseline survey (T0) just before receiving treatment. Participants who exhibited moderate depression or anxiety as determined using a Beck Depression Inventory (BDI) score of >23 or a Beck Anxiety Inventory (BAI) score of >15 during the baseline survey (T0) were also excluded. This additional screening was necessary because there was a gap between the recruitment survey and T0, and we wanted to ensure that participants with clinical levels of depression or anxiety were excluded to minimize the influence of external mental health issues on the experiment’s outcomes [50,51].

On the basis of these eligibility criteria, 85 participants were deemed suitable for evaluating the effectiveness of Moa. The participants were randomly divided into 2 groups: a treatment group comprising 55% (47/85) of the individuals, who used the Moa-integrated app, and a control group comprising 45% (38/85) of the individuals, who used the app without Moa. We used Python (Python Software Foundation) to create random allocation lists to assign participants to the treatment and control groups in a 4:3 ratio until the distribution of PHQ-9, GAD-7, PPS, and IPS scores became similar. The list generation process was repeated until the distributions were equal. Researchers were blinded to group assignment until the randomization was performed. The number of participants was powered to detect a weak effect size of 0.25 using G*Power (version 3.1.9.6; Heinrich-Heine-Universität Düsseldorf) for repeated-measure ANOVA. Assuming that 15% of the participants would leave the study by the end of the experiment given our participants’ high educational background and the incentives provided to complete the sessions [52-54], the required sample size was 82.

### Procedure

Eligible participants received a concise explanation regarding the installation of the app, use guidelines for the intervention, and the criteria for continued participation. The participants were initially asked to complete recruitment surveys that included demographic information, psychological questions such as the PHQ-9 and GAD-7, and details about their previous experiences with other time management apps and chatbots.

Participants with severe depression or anxiety, as indicated by the scores on the PHQ-9 and GAD-7, were excluded from the study. The participants were not allowed to use other to-do apps or offline journals to note their everyday tasks during the experiment. The APK file for installing the app was sent via email, and each participant signed up for the app at no cost. Participants were then randomly assigned to the treatment or control group based on the scores of their demography.

Following randomization, the participants were instructed to use the Moa intervention at least 3 or 4 times a week for a month, which was the required use period. In the treatment group, participants used the Moa-integrated to-do app, whereas the participants in the control group used the app without Moa. A push alarm reminded the participants to make lists and complete their daily tasks every morning and evening regardless of their group assignment. The only difference between the groups was the presence of a Moa message accompanying the push alarm in the evening. Participants who failed to satisfy the use requirements for a particular week were disqualified and received a reward based on the duration of their use. After the required use period, participants were allowed to use the app freely without communicating with Moa for 1 month; this was referred to as the free use period. Minor bugs in the app were fixed during the required use sessions, but no changes to the content were made.

We conducted 3 web-based assessments during the experiment: before the start of the required use period, which was immediately after the practice session (T0); after the required use period (T1); and at the end of the free use period (T2). A web-based survey was conducted to assess the overall user experience, including subjective feelings and usability, feasibility, and acceptability of and satisfaction while using the app during T1. Data on app use were automatically stored in a secure database (MongoDB; MongoDB Inc). All data obtained through the experiment were protected through anonymization.

### Measurements

#### Verification of Behavior and Cognitive Changes

The psychological scales were administered during T0, T1, and T2 to analyze any changes in the behavior of the participants during and after app use. The procrastination level of individuals was examined using the PPS (Cronbach α=0.93) and IPS (Cronbach α=0.85) [47]. The PPS typically measures general procrastination with a low likelihood of clinical problems. In contrast, the IPS measures irrational behaviors associated with mental health problems such as stress and worries. The PPS and IPS comprise 12-item and 9-item questionnaires, respectively, measured on a 5-point Likert scale. The Time Management Behavior Scale (TMBS), developed by considering the fundamental theories and strategies for time management, was used to evaluate the time management abilities of the participants (Cronbach α=0.88) [55]. Here, the factors of time management were factorized into “goal-setting and planning” (Cronbach α=0.82), “procrastination” (Cronbach α=0.86), and “practice strategies” (Cronbach α=0.86), with 9-item, 8-item, and 6-item questionnaires for each factor on a 5-point Likert scale, respectively. We used the Academic Self-Regulation Scale (ASRS) developed by the Brain and Motivation Research Institute in South Korea to identify the difference in regulative behavior with respect to academic work (Cronbach α=0.89) [56]. This scale comprises 8 items rated on a 7-point scale. Furthermore, stress, depression, and anxiety were measured to assess changes in mental status. The Perceived Stress Scale (PSS) comprises 10 items measured on a 5-point Likert scale based on 2 factors: negative perception (Cronbach α=0.77) and positive perception (Cronbach α=0.74) [57]. The PSS score was calculated by reverse-coding only the items related to positive perception among the total items, and subsequently summing the scores of all items. We used the BDI and BAI, which comprise 21 items each measured on a 4-point scale, for depression and anxiety, respectively [50,51]. The BDI and BAI were used to screen people for anxiety and depression to eliminate nuisance factors only except for measuring the intervention effect.

#### Exploration of Behavior and Cognitive Changes via Engagement

Engagement measurement tools are essential for evaluating user interaction with an app and their subsequent behavior and psychological changes [58]. Given the variety of methods for measuring engagement in digital interventions, it is essential to clearly define what specific aspect of engagement will be measured in our research [59,60]. In this context, we quantified the engagement of participants primarily based on app and chatbot engagement because the chatbot was used only by the treatment group, whereas both the control and treatment groups used the to-do app.

To obtain the detailed app engagement score, data on app use were logged each time the participants accessed the app and made entries. App engagement was measured daily using three subscores: (1) frequency of app access, (2) number of to-do lists, and (3) elapsed time of the to-do list. Each subscore was adjusted to remain in the range of 0 to 1 to ensure comparability. The daily app engagement score was obtained by summing the 3 subscores using the specifics and formulas detailed in Table S1 in Multimedia Appendix 3. These daily scores were aggregated into weekly averages, enabling the discernment and comparison of patterns across groups every week. Subsequently, we explored weekly app engagement patterns over time using k-means clustering analysis based on the correlation distance. The optimal number of clusters was determined using the elbow method. Each cluster was then characterized according to its unique engagement pattern over time. Furthermore, we evaluated the changes in behavioral and psychological scores within each of these clusters for each group.

To verify the effects of the chatbot intervention, we calculated the chatbot engagement score exclusively for the treatment group. This score incorporated several elements, including the length and duration of user responses and emotional scores derived from conversations with Moa. Emotional scores ranging from 0 (indicating negative emotions) to 1 (indicating positive emotions) were extracted using the Korean Bidirectional Encoder Representations from Transformers sentiment classifier; simple responses such as “yes” or “no” were excluded from emotional scoring. Considering the variability in the quality and content of user responses required in scenario-based chatbot conversations, temporal changes in chatbot engagement were not considered in our analyses. By comparing the chatbot engagement scores across different app engagement clusters, we aimed to elucidate the potential influence of chatbot interventions on behavioral modification.

#### Exploration of User Experience

User experience was quantified via the user test surveys. The usability of the developed apps was measured using the 10-item System Usability Scale [61] on a 5-point Likert scale. The total score was calculated by multiplying 2.5 by the sum of the item scores. The standards for the total scores were “OK if >55,” “good if >75,” and “excellent if >87.5” [62]. The acceptability of the app, management of procrastination, and feasibility were assessed considering the Usage Rating Profile–Intervention (URP-I) [63]. The URP-I was designed to measure the factors that individuals adopt and subsequently use in the intervention over time. We used the 8-item subscale associated with acceptability and the 6-item subscale for feasibility in the URP-I, adapting the questionnaires to the purpose of our app using a 6-point Likert scale. The general satisfaction with the intervention in the treatment and control groups was assessed using an 8-item client satisfaction questionnaire measured on a 4-point Likert scale [64].

User experience in the treatment and control groups was assessed based on the overall feeling of the users when using the app, collected via a subjective questionnaire (“Please write the overall feeling freely after using the app”). Participants were given additional explanations of the questions to guide their responses regarding aspects such as the advantages and disadvantages, similarities to prior expectations, and any behavioral changes they experienced.

### Analytic Strategy

To analyze the self-reported surveys, linear mixed models were fitted for repeated measures of each feature investigated in the questionnaires (ie, procrastination, time management, academic self-regulation, and stress). We critically considered 3 effects: the effect of the chatbot intervention by comparing the treatment and control groups (*group*), the effect of time (T0, T1, and T2; *time*), and the interaction of *group* and *time*. Both *group* and *time* were nominalized and set as fixed effects. The interaction effect of *group* and *time* was set as an effect of the model to explain how chatbots impact behavior or cognitive changes. We set the score of each scale at T0 as a covariate to control for the initial difference between the 2 groups. Participants considered as random effects in the model were nested into the *group* effect. A linear mixed model was fitted using the RStudio (version 4.3; Posit PBC) *nlme* package [65]. We conducted post hoc pairwise comparisons considering different numbers of samples from each group using the Scheffé method with the *emmeans* library in RStudio to conservatively determine the primary effect [66,67].

In the clusters derived from the temporal patterns of app engagement, the objective was to compare the changes in questionnaire scores obtained from T0 to T1 across each group. The sample sizes were reduced because of cluster division, necessitating nonparametric testing. Consequently, we used a 2-sided Wilcoxon signed-rank test in our analyses. In addition, separate tests were conducted for the 2 groups (treatment and control) and 3 clusters (high, mid, and low) within each questionnaire, resulting in a total of 6 comparisons. To control for the risk of type-I error due to multiple comparisons, we applied a Bonferroni correction. To investigate the differences in chatbot engagement scores among the clusters, we conducted the Kruskal-Wallis ANOVA test followed by the Scheffé post hoc test for multiple cluster comparisons.

To account for the potential influence of participants who did not consistently engage with the intervention, we performed an intention-to-treat analysis initially. In this case, we filled out the missing values for withdrawals by using the scores that were previously measured during the prior assessment. In addition, we conducted a completer-only analysis excluding participants who withdrew during the required use period (T1) to assess the effect of the intervention while controlling more precisely for nuisance variables such as use frequency. A post hoc test was subsequently conducted on the completer data.

To quantitatively analyze the user experience, we calculated the total score of each test survey to examine user experience through simple statistics. The difference between the treatment and control groups was validated via a proportion *z* test using positive survey results. For the review data, we conducted a thematic analysis to elucidate the findings derived from subjective responses using QDA Miner Lite (Provalis Research). The smallest level, features of the intervention, was identified from the users’ reviews, and the responses with similar features were combined to form codes. They were categorized into 2 groups: the intervention effect that individuals can experience cognitively and the functions that individuals mentioned positively or negatively. The functions of the intervention were further categorized into graphical UI–based to-do app and chatbot-based to-do app. The coding of the data was discussed with 3 coauthors to ensure accuracy, and the codes were refined by carefully reviewing the content.

Finally, we calculated the percentage of sentences that were coded with codes from the intervention effect and positive function categories together to explore relationships between the functions and the effect that users experience. If a code from the intervention effect category co-occurred with one from the positive function category in ≥40% of the sentences, they were assumed to be related.

All statistical analyses were conducted using RStudio (version 4.3) [68].

## Results

### Participant Characteristics

**Figure 3:**
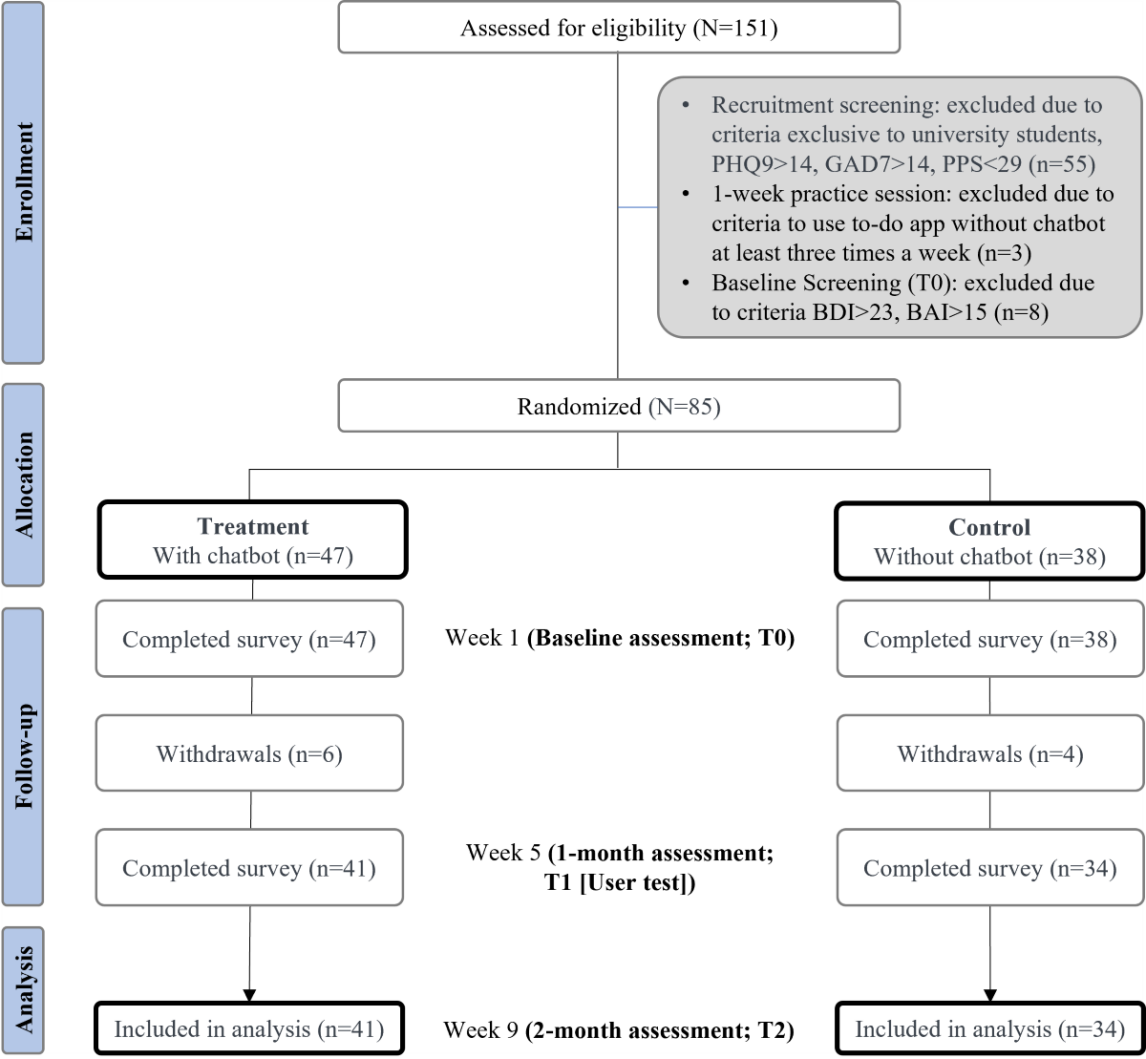
Study flowchart representing randomization and criteria to exclude participants throughout the study. BAI: Beck Anxiety Inventory; BDI: Beck Depression Inventory; GAD-7: Generalized Anxiety Disorder–7; PHQ-9: Patient Health Questionnaire–9; PPS: Pure Procrastination Scale.

We recruited participants starting in February 2023 and completed our experiment in May 2023. Initially, 85 participants were eligible for inclusion in the study. Participants were randomly divided into 2 groups based on their balanced characteristics. We validated the balanced distribution of the demographic, psychological, or behavioral characteristics using a 2-tailed *t* test. Table 1 summarizes the detailed information about the participants. Figure 3 shows the withdrawal of participants based on the inclusion and exclusion criteria.

**Table 1:** Participants’ demographics.

Characteristics		Treatment (n=47)	Control (n=38)	*P* value^a^
**Demographic characteristics**				
	Age (y), mean (SD)	20.6 (2.0)	20.2 (1.9)	.29
	Sex (female), n (%)	19 (40)	18 (47)	—^b^
**Baseline psychological and behavioral characteristics, mean (SD)**				
	PHQ-9^c^ score	3.6 (3.2)	4.4 (3.5)	.26
	GAD-7^d^ score	2.6 (2.8)	2.8 (2.7)	.75
	PPS^e^ score	42.9 (6.7)	40.6 (6.8)	.13
	IPS^f^ score	32.2 (5.4)	31.4 (4.9)	.48

^a^*P* values are the result of 2-sided *t* tests.

^b^*t* test results are not available for sex as it is a binary variable.

^c^PHQ-9: Patient Health Questionnaire–9.

^d^GAD-7: Generalized Anxiety Disorder–7.

^e^PPS: Pure Procrastination Scale.

^f^IPS: Irrational Procrastination Scale.

### Verification of Behavior and Cognitive Changes

Table 2 summarizes the results of the *F* test conducted via the linear mixed model using the data from T0 to T1 and the differences between these time points using the intention-to-treat method. The results indicate that the absolute value of the difference in the treatment group was generally higher than that in the control group. The interaction of *time* × *group* was significant in the procrastination subscale of the TMBS (*P*=.03). The *time* effect on all questionnaires except for “Goal-setting/planning,” which is a subscale of the TMBS and ASRS, was significant (*P*<.001), whereas the *group* effect was not.

**Table 2:** Difference between T0 and T1 with F statistic with a linear mixed model setting scores at T0 as a covariate (intention-to-treat analysis).

			T1–T0, mean (SD)				*F* test (*df*)							*P* value				
			Treatment (n=47)		Control (n=38)		Time		Group		Interaction		Time			Group		Interaction
Pure Procrastination Scale			−10.29 (7.74)		−7.68 (6.93)		103.6 (1, 83)		4.05 (1, 82)		1.08 (1, 83)		<.001			.048		.30
Irrational Procrastination Scale			−4.54 (5.59)		−2.53 (6.13)		40.44 (1, 83)		2.04 (1, 82)		2.7 (1, 83)		<.001			.16		.10
**Time Management Behavior Scale**			7.56 (10.6)		4.09 (9.69)		30.46 (1, 83)		0.09 (1, 82)		1.98 (1, 83)		<.001			.77		.16
	Goal setting/planning	0.49 (4.71)		−0.68 (4.62)		1.77 (1, 83)		1.01 (1, 82)		1.01 (1, 83)		.19			.32		.32	
	Procrastination	4.61 (4.47)		2.24 (4.06)		4.74 (1, 83)		4.77 (1, 82)		4.77 (1, 83)		.03			.03		.03	
	Practice strategies	2.46 (4.59)		2.53 (4.39)		2.33 (1, 83)		0.01 (1, 82)		0.01 (1, 83)		.13			.92		.92	
Academic Self-Regulation Scale			−0.12 (6.12)		−0.21 (6.24)		0.13 (1, 83)		7.69 (1, 82)		0.04 (1, 83)		.72			.007		.84
Perceived Stress Scale			−2.46 (4.64)		−1.38 (4.63)		19.37 (1, 83)		0.03 (1, 82)		1.05 (1, 83)		<.001			.87		.30

The results from a linear mixed model from T0 to T1 to T2, which included a month after the end of the required app use period, revealed that the *time* effect was significant at every scale (*P*<.001).

We also analyzed the completers excluding participants who withdrew from the study due to insufficient app use. The results are presented in Table S3 in Multimedia Appendix 3. With the exclusion of 12% (10/85) of the participants, the overall *P* value decreased slightly. Notably, the interaction of the procrastination subscale of the TMBS showed significant results (*P*=.01). In addition, irrational procrastination measured using the IPS was close to the significance level (*P*=.07).

An overview of each questionnaire examined at the time points before and after the required app use period (T0 and T1) and after a month (T2) is provided in Multimedia Appendix 3 with 95% CIs. Most participants exhibited improved scores for all variables except the ASRS.

**Figure 4:**
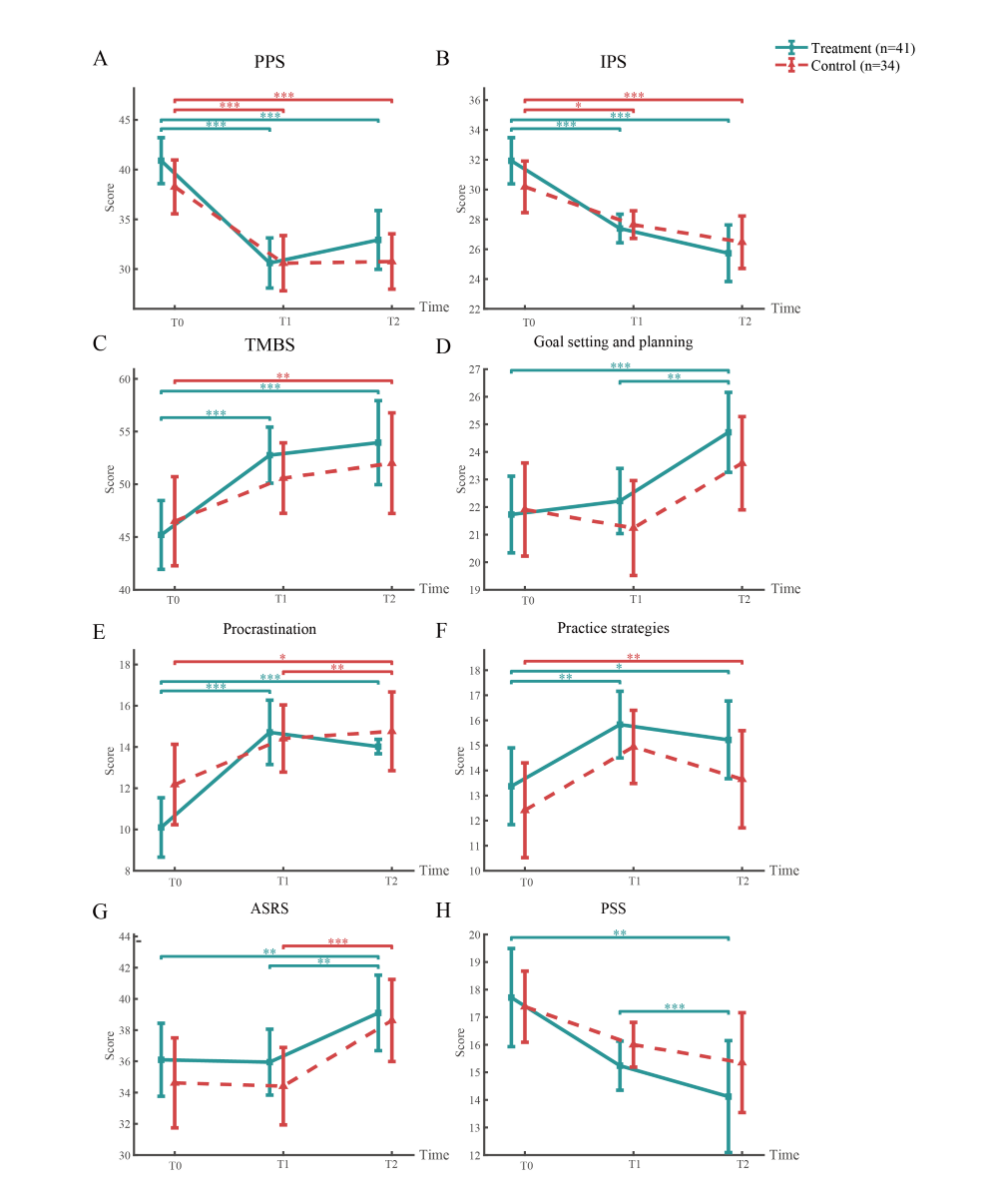
Changes from T0 to T1 to T2 in the questionnaire scores with significance from the Scheffé test among completers (mean and 95% CI). *P<.05, **P<.01, ***P<.001. ASRS: Academic Self-Regulation Scale; IPS: Irrational Procrastination Scale; PPS: Pure Procrastination Scale; PSS: Perceived Stress Scale; TMBS: Time Management Behavior Scale.

We conducted a post hoc test using the Scheffé method to identify behavioral and psychological change within each group among completers. Figure 4 illustrates the scale variations, indicating the significance level based on the results of the Scheffé test. As indicated in the figure, the PPS scores decreased in both the treatment and control groups regardless of the use of Moa. Conversely, irrational procrastination measured using the IPS exhibited a strong significant tendency to decline in the treatment group between T0 and T1 (mean −4.54, SD 5.59, 95% CI −6.30 to −2.77; *P*<.001) and between T0 and T2 (mean −6.20, SD 5.78, 95% CI −8.02 to −4.37; *P*<.001), whereas the control group showed a smaller significant variation between T0 and T1 (mean −2.53, SD 6.13, 95% CI −4.67 to −0.39; *P*=.02).

The TMBS score, which measures time management, increased significantly from T0 to T1 (mean 7.56, SD 10.6, 95% CI 4.22-10.91; *P*<.001) and maintained the intervention effect until T2 in the treatment group; however, it was not statistically significant in the control group (mean 4.09, SD 9.69, 95% CI 0.71-7.47; *P*=.05). The control group did not exhibit significant changes between T0 and T2 in the “Goal-setting/planning” subscale (mean 1.68, SD 4.54, 95% CI 0.09-3.26; *P*=.07). In contrast, the treatment group exhibited a significant increase in scores from T0 to T2 (mean 2.98, SD 4.93, 95% CI 1.42-4.53; *P*<.001).

The PSS scores decreased over time regardless of the group, but only the treatment group showed a significant change. Remarkably, the treatment group exhibited a significant difference in PSS scores between T0 and T2 (mean −3.59, SD 6, 95% CI −5.48 to −1.69; *P*<.001).

The effect of *time* between T0 and T1 was insignificant on the ASRS; however, the difference between T0 and T2 (treatment: *P*=.003; control: *P*<.001) and between T1 and T2 (treatment: *P*=.002; control: *P*<.001) was significant.

### Exploration of Behavior and Cognitive Changes via Engagement

**Figure 5:**
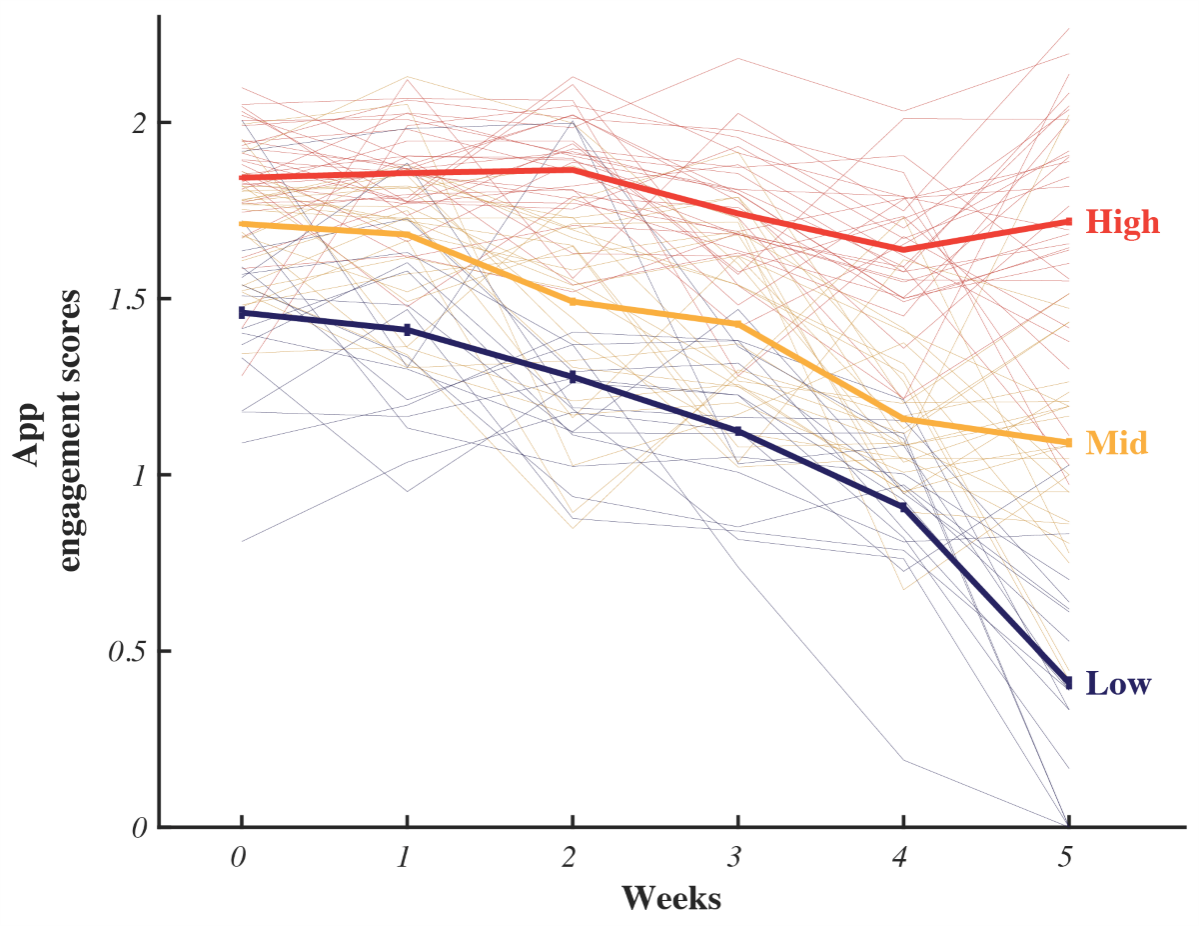
Temporal changes in app engagement scores within each cluster (mean and 95% CI).

We compared the patterns of temporal change in app engagement scores between the treatment and control groups (Table S4 in Multimedia Appendix 3). Although the results of the *F* test showed that both groups exhibited a decrease in app engagement scores over time (*F*_5, 355_=43.44; *P*<.001), no interaction effect existed between *time* and *group* (*F*_5, 365_=0.36; *P*=.94). Therefore, we extracted clusters of app engagement changes over time based on correlation distance–based k-means clustering. The optimal number of clusters, determined using the elbow method, was 3 (Figure S3 in Multimedia Appendix 3). We categorized users into 3 clusters: “high,” “middle,” and “low,” representing their level of sustained engagement with the app during the intervention (Figure 5). In the high cluster, users maintained their engagement scores relatively consistent throughout the practice (week 0) and required use (weeks 1-5) periods in comparison with the middle and low clusters. Although the app engagement scores of both the middle and low clusters decreased over time, the scores of the middle cluster remained higher than those of the low cluster, and the scores of the high cluster remained significantly higher than those of the middle cluster at all time points (Tables S5 and S6 in Multimedia Appendix 3).

We observed changes in the behavioral assessment results corresponding to variations in app engagement. Table 3 lists these changes by presenting the differences in questionnaire scores between T0 and T1 per cluster within each group. Notably, the findings mirror those in Table 2, indicating an overall score improvement across the groups. The Bonferroni-corrected *P* values for each of the 6 comparisons (treatment: high, mid, and low engagement; control: high, mid, and low engagement) are also reported in Table 3, reflecting the significance of these changes after adjusting for multiple comparisons.

**Table 3:** Descriptive statistics and Wilcoxon signed-rank test for behavioral and psychological assessment—comparing T1 and T0 within groups in each cluster.

			Changes during the required use period (T1–T0), mean (SD)											
			Treatment							Control				
			High engagement (n=13)		Mid engagement (n=15)		Low engagement (n=13)		High engagement (n=11)			Mid engagement (n=12)		Low engagement (n=11)
PPS^a^			−10.7^b^ (6.5)		−9.4^c^ (7.7)		−10.8^c^ (9.9)		−8^c^ (5.7)			−7.5^c^ (7.4)		−7.4 (9.0)
IPS^d^			−4^c^ (4.8)		−5.1 (5.1)		−4.7 (7.1)		−3 (7.2)			−0.8 (5.2)		−5.1 (5.4)
**TMBS^e^**			5.9 (8.3)		7.8 (11.2)		9.7 (13.4)		8.9 (10.8)			1.9 (7.8)		−0.4 (8.1)
	Goal setting/planning	−0.1 (4.7)		0.2 (3.9)		1.6 (5.7)		1.8 (4.5)			−2.1 (4.6)		−2.6 (2.7)	
	Procrastination	4.9^b^ (3.7)		4.8^c^ (4.8)		3.8 (5.5)		3.5 (3.7)			2.1 (3.7)		0.3 (5.1)	
	Practice strategies	1.1 (3.9)		2.8 (5.0)		4.3 (4.8)		3.6 (4.8)			1.9 (3.3)		0.3 (5.6)	
ASRS^f^			−1.3 (5.7)		0.4 (7.5)		1.1 (5.1)		2.1 (6.3)			−1.6 (6.1)		−1.6 (6.0)
PSS^g^			−2.7 (4.1)		−3.2 (4.7)		−1.3 (5.5)		−1.7 (4.1)			−0.9 (5.0)		−1.7 (5.3)

^a^PPS: Pure Procrastination Scale.

^b^The statistics are significant at *P*<.001 after Bonferroni correction.

^c^The statistics for the 2-sided Wilcoxon signed-rank test comparing the scores at T1 and T0 are significant at *P*<.01 after Bonferroni correction.

^d^IPS: Irrational Procrastination Scale.

^e^TMBS: Time Management Behavior Scale.

^f^ASRS: Academic Self-Regulation Scale.

^g^PSS: Perceived Stress Scale.

The PPS scores revealed a significant difference in all clusters within the treatment group and all except for the low cluster within the control group. Even after applying the Bonferroni correction, this difference remained significant in every cluster of the treatment group (high: *P*=.004; middle: *P*=.01; low: *P*=.002) and in the high (*P*=.01) and middle (*P*=.03) clusters of the control group. The IPS scores indicated a significant difference in the high and middle clusters of the treatment group only, which remained significant in the high cluster (*P*=.046) after correction.

We examined the Procrastination subscale scores on the TMBS and found significant differences in all clusters within the treatment group; however, only the high cluster was significant in the control group. After Bonferroni correction, the high (*P*=.004) and middle (*P*=.02) clusters of the treatment group remained significant.

The analysis of app engagement scores revealed that, unlike the control group, the treatment group exhibited a continued intervention effect even in the middle cluster, with decreased app engagement over time.

**Figure 6:**
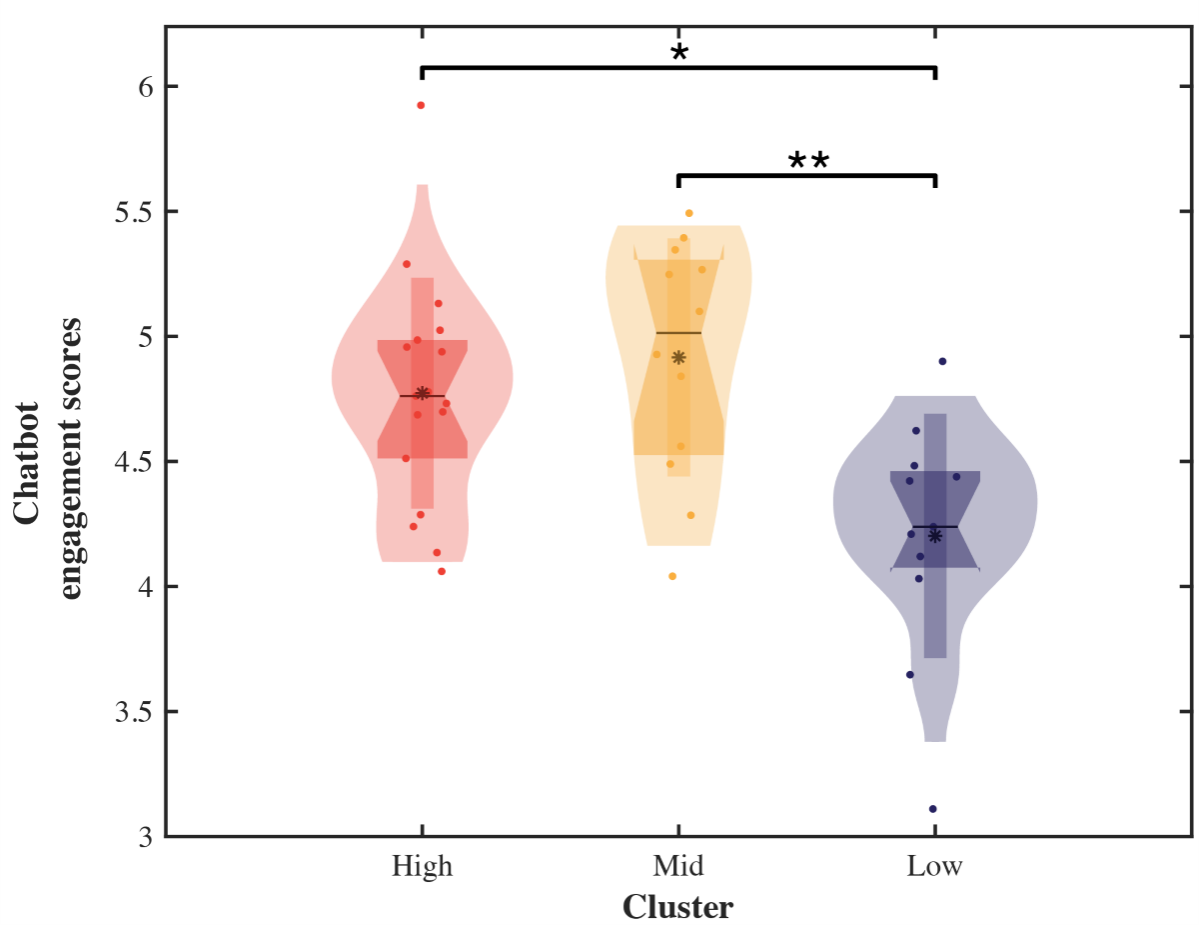
Comparison of chatbot engagement scores between clusters.

The analysis of app engagement indicated that even users with relatively low app engagement experienced behavior changes under the treatment conditions, unlike what was observed in the control group. Therefore, we aimed to elucidate these effects through an engagement analysis of the Moa chatbot provided in the treatment group based on app engagement score cluster. The chatbot engagement scores exhibited significant differences between the clusters (H2=10.79; *P*=.005; Kruskal-Wallis ANOVA test). Following a Dunn post hoc analysis, the high and low (*P*=.02) and middle and low (*P*=.006) clusters demonstrated significant differences in chatbot engagement scores between them. Conversely, the disparities between the high and middle clusters were not statistically significant (Figure 6). The significantly higher chatbot engagement scores in the middle cluster compared with those of the low cluster support the possibility that significant behavior changes occurred in the users in the middle cluster in the treatment group, unlike in those from the control group.

### Exploration of User Experience

We conducted surveys to test the usability, feasibility, and acceptability of and satisfaction with the intervention in each group (details are presented in Multimedia Appendix 3). All participants who completed the psychological questionnaires also took part in the user experience survey (n=75). Our analysis determined that >70% of the participants (58/75, 77%) scored above the midpoint of the Likert scale in every dimension of the user test. The average usability score assessed using the System Usability Scale was 72.8 (SD 16.0) in the treatment group and 75.15 (SD 10.1) in the control group, indicating that the intervention was “good” to use regardless of the presence of Moa [61]. No significant differences were observed between the control and treatment groups in terms of usability, feasibility, and satisfaction. However, participants in the treatment group exhibited significantly better acceptability than those in the control group (*P*=.03).

Table 4 summarizes the results of the thematic analysis of the qualitative responses regarding the app use experience of the participants in each group. The contents were categorized as intervention effects on behavioral and cognitive improvements and positive and negative features of the intervention.

**Table 4:** Results of the thematic analysis.

Theme and codes			Cases, n (%)	
			Treatment (n=41)	Control (n=34)
**Intervention effect on the user**				
	**Superficial effect**			
		Take action for today	11 (27)	7 (21)
		Preparation to do	8 (20)	9 (26)
		Review what the user did	5 (12)	2 (6)
	**Inner effect**			
		Diligence and sincerity	16 (39)	6 (18)
		Motivation	12 (29)	6 (18)
		Self-reflection	5 (12)	1 (3)
		Alliance	9 (22)	0 (0)
**Positive features of...**				
	**To-do app**			
		Function for to-do list up	8 (20)	18 (53)
		Simple	5 (12)	2 (6)
		Alarm	2 (5)	6 (18)
		Calendar	3 (7)	3 (9)
	**Moa augmentation**			
		Advice or feedback	11 (27)	—^a^
		Consolation or cheering utterance	7 (17)	—
		Conversation for reflection	5 (12)	—
		Caring	4 (10)	—
		Friendly tone	2 (5)	—
**Negative features of...**				
	**To-do app**			
		Fixed time to finish work	4 (10)	7 (21)
		Lack of function	5 (12)	7 (21)
		System error	3 (7)	5 (15)
		Inconvenient UI^b^	4 (10)	2 (6)
	**Moa augmentation**			
		Illogical answers	16 (39)	—
		General feedback or not interesting	4 (10)	—

^a^The control group did not interact with the chatbot, resulting in missing values for this variable.

^b^UI: user interface.

The effects of the intervention on the users were divided into superficial and inner effects. The superficial effect theme included 3 codes: *Take an action for today*, *Preparation to do*, and *Review what the user did*. The answers that facilitated task performance, such as “I was able to perform well without forgetting things” and “Writing things down in advance, I have reduced the number of missed assignments,” were coded as *Take an action for today*. We observed that participants could plan and envision their tasks (*Preparation to do*) based on responses such as “I could check what to do tomorrow” and “I liked being able to think and organize my tasks every morning.” Several participants reviewed their tasks as follows: “It was great having time to review the plans I had set and what I’ve done that day,” which was coded as *Review what the user did.* The percentage of the cases in the treatment and control groups was not remarkably different except for *Review what the user did*, which was mentioned by 12% (5/41) of the participants in the treatment group and only 6% (2/34) of the participants in the control group. These effects were considered superficial changes because they were directly associated with the to-do functions, including establishing lists of tasks to execute, checking the tasks completed by users, and visually displaying these to the users.

The inner effect did not directly vary according to the functions of the app and was developed internally using traits and mindsets. We identified *Diligence and sincerity*, *Motivation*, *Self-reflection*, and *Alliance* as inner effects. Participants who experienced an improvement in their procrastination habits or enhanced sincerity answering “I have significantly reduced the habit of procrastinating on tasks” and “I lead a more fulfilling life than before” were coded as *Diligence and sincerity*. Participants coded as *Motivation* were motivated to plan or execute their tasks, and stated the following: “(Moa) gave me more determination to stick to my plans” and “Looking at the lists, I was able to receive the spirit of challenge that encourages to put things into practice.” Several participants reflected on their habits and goals, indicated by the following statements: “I could notice my procrastination pattern” and “I started to contemplate how I should live my day,” which were classified as *Self-reflection*. In comparison with the control group, more participants in the treatment group experienced internal changes. Only the participants in the treatment group perceived the app as a mode of communication rather than as a mere tool, enabling them to gain internal support that represented an *Alliance* between the users and Moa; this was indicated by statements such as the following: “Sometimes, Moa became a source of comfort” and “Feeling like having a friend who constantly takes care of my schedule.”

There were functions that were evaluated positively by the users. Participants evaluated the *Function for to-do list* as helpful, saying the following:

By obligingly jotting down today’s tasks, I could lead a more fulfilling life.

The simple UI of the app was positively evaluated (eg, “UI is neat, and app usage is intuitive” [*Simple*]). Although the treatment group’s app contained more features and was more complicated to use, there were more responses coded as *Simple* than in the control group. The alarm function coded as *Alarm* was considered positive, indicated by statements such as the following:

Getting daily reminders helped me develop a good habit.

Participants who evaluated *Calendar* as suitable visual feedback mentioned the following:

It was also great to see my achievements at a glance through the calendar.

Among the positive aspects after Moa augmentation, over one-fourth of the participants (11/41, 27%) mentioned that *Advice/feedback* from Moa was a positive feature, saying the following:

During conversations with Moa, I learned various (time management) tips.

Similarly, 12% (5/41) of the participants, who responded, “I enjoyed having the opportunity to think about things I don’t usually consider and reflect on overlooked aspects while chatting with Moa,” received support from *Conversation for reflection*. In addition, *Consolation/cheering utterance* was considered a positive aspect, indicated by statements such as the following:

I appreciated having someone in Moa who could cheer me on and support my plans during our conversations.

*Caring* was also one of the positive aspects of the chatbot, indicated by statements such as the following:

...I received confirmation through the chatbot, the success rate of my plans increased.

Responses such as the following—“The cute and friendly tone made me feel comfortable, allowing me to have conversations with Moa with ease”—were coded as *Friendly tone*.

The app had certain negative aspects as well. Participants were uncomfortable with the *fixed time to finish the work*, which was set to 11 PM in the experiment. They commented the following:

Sometimes, I have experienced forgetting to check and press the “Finish” button when a planned task extends beyond midnight.

Several participants criticized that the app had a *lack of functions* during use:

I often find it inconvenient that I have tasks that frequently extend well beyond the 11 p.m. deadline.

Participants who complained about the errors were coded as *System error*. In contrast to the opinion that the UI was simple and intuitive, several participants evaluated it by saying the following—“it seems that the convenience of the to-do list up feature has somewhat declined, possibly due to a focus on the Moa”—indicating an *Inconvenient UI* of the app.

Furthermore, 39% (16/41) of the participants conversing with Moa experienced *illogical answers*, indicated by statements such as the following: “Sometimes, artificial intelligence can speak awkwardly” or “I felt a bit frustrated while conversing with Moa.” Additional opinions mentioned the following: “It seems Moa’s instructions are too generic, so it doesn’t provide much help” or “The responses were more limited than expected, and the chatbot’s lack of understanding made the conversation less enjoyable.”

The codes from *intervention effect* that were present along with *positive features* were analyzed to identify the relationship between functions of the app and associated effects.

The codes for superficial effects did not have a significant relationship with other positive features in the treatment group. In the case of the control group, 86% (6/7) and 67% (6/9) of the codes associated with *Take an action for today* and *Preparation to do* appeared with *Function for to-do list up*, respectively.

Over 64% of the participants who experienced the inner effects of *Motivation* and *Diligence and sincerity* in the control group (7/11) mentioned *Function for to-do list up* as a positive feature of the app.

In contrast, the treatment group participants tended to touch the features of the Moa chatbot more than the elements of the to-do app with inner effects. For instance, 44% (7/16) of the people who experienced more *Diligence and sincerity* mentioned the *Advice/feedback* of the chatbot as a positive feature. One participant stated the following:

I found it fascinating that the app taught me how to plan and stick to my schedule, and my procrastination habit has significantly reduced in practice.

Furthermore, 80% (4/5) and 60% (3/5) of the participants underwent *Self-reflection* based on *Conversation for reflection* and *Advice/feedback,* respectively. One participant stated the following:

The best part was being able to receive feedback and reflect while making plans through conversations with Moa.

The *Consolation/cheering utterance* of Moa appeared with *Motivation* and *Alliance* with an occurrence of 42% (5/12) and 67% (6/9), respectively. Remarkably, 44% (4/9) of the participants who mentioned *Alliance* as the effect of the app considered *Management* as a positive feature of the chatbot, indicated by statements such as the following:

Unlike other apps, Moa monitors my behavior and supports me, which motivated me to participate more actively.

## Discussion

### Principal Findings

In this study, we developed a CBT-based semigenerative chatbot named Moa and integrated it with a to-do list app for time management. To validate its effectiveness and usability, we designed a graphical UI–based to-do list app with the features of planning, checking tasks, and monitoring. The control group used the app without Moa, whereas the treatment group used the app with the chatbot, receiving CBT sessions for 5 weeks.

We demonstrated the necessity of integrating the chatbot with the to-do app to provide further assistance in facilitating behavior changes, planning, and monitoring. In comparison with the control group, the treatment group exhibited higher variations in time management and procrastination scale scores. Notably, individuals who were not used to maintaining to-do lists yet sincerely communicated with the chatbot reported a significant reduction in procrastination. Conversely, little or no change was observed in the control group unless the participants continued using the to-do list consistently. This result confirms that a to-do app with Moa can instigate behavior changes, particularly for people not experienced in managing tasks. In the treatment group, a higher proportion of individuals subjectively perceived reduced procrastination habits compared to the control group. In addition, according to the results of the thematic analysis, the chatbot provided self-reflection opportunities and emotional support, which are not offered by apps without a chatbot.

Procrastination, rooted in the “avoidance of the implementation of an intention” [69], has multiple underlying causes, including poor time management, pressure-seeking behavior, anxiety, and perfectionism [70]. Although improved time management can mediate procrastination, it does not serve as a comprehensive treatment for it. The chatbot, built on the offline academic procrastination CBT process, offered tailored advice and feedback based on the various causes of procrastination. This principle enabled the chatbot to intervene effectively by following the different procrastination factors for each individual.

The effectiveness of the chatbot originated from the possibility of Moa to provide emotional assistance while forming an alliance with users based on their reviews. The variation in scores for irrational procrastination and stress in the treatment group was significantly greater than that in the control group compared with the other scales from the ANOVA result. Although conversing with Moa cannot equal interacting with humans, users experienced emotional interventions by feeling cared for and supported. This indicated that engaging in conversations with the developed chatbot enabled emotional interventions to influence stress relief and emotional regulation, which are critical for treating irrational delaying behaviors [71].

Despite the addition of the chatbot, the app was evaluated as simple and feasible to use. The only difference was in acceptability, which was higher in the treatment group than in the control group.

### Strengths and Limitations

Considering that most time management apps focus on productivity and organizing tasks rather than on the psychological concept of procrastination [7-9,72], this study leveraged a psychological approach by combining CBT with a chatbot to directly address procrastination. The developed chatbot not only addressed procrastination as a behavioral issue but also considered various causes such as stress and perfectionism, thereby enabling tailored feedback. Moreover, it could effectively handle procrastination behaviors grounded in the offline counseling process and reviews provided by counselors and psychologists.

Our findings confirmed that the chatbot not only accelerates behavior changes but also significantly impacts the psychological aspects. This was achieved by fostering introspection and providing valuable support and advice to users, which enabled them to cultivate a sense of connection with the chatbot. These results indicate that chatbots can address procrastination issues from a broader perspective than traditional time management apps.

Although generative chatbots enable natural conversations, they are less suitable for goal-oriented conversations because the overall conversation content can vary based on user utterances [30]. Furthermore, users may experience critical confusion if chatbots respond inappropriately. However, the developed semigenerative chatbot ensured a consistent dialogue within a predefined framework, allowing it to retain the direction of the conversation even when several responses were not entirely reasonable. In other words, the semigenerative chatbot enabled users to engage in relatively natural conversations while maintaining a consistent dialogue flow.

In this study, the control group was allocated to use a to-do app without the chatbot rather than assigning the participants to a waitlist. This facilitated the determination of distinct characteristics of the chatbot compared with a basic time management app. One perspective suggests that using a to-do app on its own can enable individuals to develop time management skills, establish habits, and initiate behavior changes. Therefore, we aimed to investigate the additional effects of using a chatbot when combined with existing apps. This was in contrast to typical validation studies on new interventions because the proposed approach assessed the benefits of incorporating a chatbot into an existing time management app from various perspectives. Rather than relying solely on self-report questionnaires to compare the treatment and control groups, we conducted a comprehensive analysis of the impact of the chatbot by clustering intervention engagement and overall user experience. These additional analyses provided a detailed understanding of the advantages of chatbot integration. Our study provides practical insights that can be applied to real-world scenarios, facilitating the adoption of chatbot technology in future studies and apps.

We observed the change from before to immediately after the intervention and validated that the effectiveness of the chatbot was prolonged even after the users completed conversing with the chatbot.

However, several limitations were observed in the analysis. Although the intervention effectively changed the behavior of users and received an excellent evaluation in the user test, participants reported troublesome unusual responses because of the fine-tuning of the model using limited data. Continuous errors may affect the satisfaction, trustworthiness, and communicative effectiveness of the app. Therefore, the model performance must be improved to reduce errors. In addition, the finite number of chatbot sessions poses a challenge for sustained use, suggesting the necessity for additional research to enable prolonged engagement. Moreover, participants were obligatorily advised to use the app at least 3 times per week for experimental purposes. However, the feasibility of consistent use without such obligations remains uncertain. Therefore, further research is required in terms of app use in unconstrained environments.

Finally, our target population consisted of university students without severe illnesses, limiting the generalizability of the findings to a broader demographic. To enhance generalizability, future studies should extend their scope to include diverse populations across various age groups and psychopathological conditions.

### Conclusions

We developed Moa, a semigenerative chatbot based on the CBT process, to address procrastination and support the behavior changes intended to be derived from time management apps. This study demonstrated that integrating a chatbot into a basic time management app can effectively change user behavior and reduce procrastination based on an RCT. The chatbot addresses procrastination as more than a simple time management issue, recognizing it as a complex interplay of various psychological and behavioral factors. This trial suggests that chatbot intervention can provide additional psychological assistance, such as fostering introspection, forming an alliance with Moa, and providing support and advice, which are not offered by a basic time management app. However, as this study represents the initial stages of chatbot development and implementation, further improvement is necessary in chatbot performance and app functionality.

## Appendix

Multimedia Appendix 1 Moa scenario content.

Multimedia Appendix 2 The 6 factors of procrastination—results of the literature review.

Multimedia Appendix 3 Supplementary information, tables, and figures for the experimental results.

Multimedia Appendix 4 CONSORT-eHEALTH checklist (V 1.6.1).

## Abbreviations

ASRS: Academic Self-Regulation Scale
BAI: Beck Anxiety Inventory
BDI: Beck Depression Inventory
CBT: cognitive behavioral therapy
GAD-7: Generalized Anxiety Disorder–7
IPS: Irrational Procrastination Scale
PHQ-9: Patient Health Questionnaire–9
PPS: Pure Procrastination Scale
PSS: Perceived Stress Scale
RCT: randomized controlled trial
TMBS: Time Management Behavior Scale
UI: user interface
URP-I: Usage Rating Profile–Intervention

## Data Availability

The datasets generated or analyzed during this study are not publicly available due to participant confidentiality agreements but are available from the corresponding author on reasonable request.
