# Supplementary material for: Development of a Mobile Intervention for Procrastination Augmented With a Semigenerative Chatbot for University Students: Pilot Randomized Controlled Trial
Source: JMIR Mhealth Uhealth. 2025 Apr 10;13:e53133. doi: 10.2196/53133 (PMC12022524; doi:10.2196/53133)
Supplement: Multimedia Appendix 1 [file mhealth_v13i1e53133_app1.pdf]

Appendix 2. Moa Scenario Contents

Session	Activities	Conversation example
Session 1	Primary conversation for a day: Observing oneself<br>➢ Asking about hobby, likes, careers and etc..<br>Short conversation for 3 days<br>➢ The reason why they finish/do not finish their work following the achievement rate at the end of the day	MOA : What is the life style that you want to have?<br>User : A life where I can travel leisurely<br><br>MOA: What factors that make you delay the tasks?<br>User: Never experiencing significant issues due to procrastination or overestimating one's own abilities.
Session 2	Primary conversation for a day: Understanding strengths and weakness while working<br>➢ Asking about strength, routines<br>➢ Noticing one's primary factor to procrastinate classified by procrastination factor algorithms<br>➢ Setting the long- and short-term goals<br>Short conversation for 3 days<br>➢ Giving applause or advice according to the achievement rate at the end of the day	MOA : What is your personal motivation when you work?<br>User : I feel like life becomes more vibrant when you work efficiently and also make time to have fun!<br><br>(When users achievement rate is low)<br>MOA: Have you ever procrastinated just because you felt lazy or for no particular reason? Let's try setting up a specific plan for each day. Create a schedule in hourly increments and use a checklist to track whether you've completed your tasks. How does that sound?
Session 3	Primary conversation for a day: Reflection of one's behavior for a last week<br>➢ Asking for the situation that they do the tasks punctually, how they thought or felt at that time<br>➢ Asking for the situation that they do delay the tasks, how they thought or felt at that time<br>Short conversation for 3 days: Setting one's strategies and take an action with MOA<br>➢ Sharing one's strategies not to delay the tasks with MOA and hold one's mind to act	MOA : Think about a time in the past week when you successfully avoided procrastination and followed through with your tasks. Please provide detailed information about that situation.<br>User :<br>I didn't procrastinate when I had somewhere important to go the next day; I completed everything on that day.<br><br>MOA : We had devised strategies to boost our goal achievement, remember? My goal was to wake up at 9 AM, what was yours? If you're ready to say it, say "yes" and if you can't recall it well, say "No"<br>User : Yes<br>MOA : What was that?<br>User : Establishing a regular sleep schedule
Session 4	Primary conversation for a day: Managing fear<br>➢ Letting users to know one of the primary psychological cause-fear and how to deal with it<br>Short conversation for 3 days: Effective strategies to manage time<br>➢ Letting users to know effective time management skills that can be used to plan for a day	MOA : Hello! Today, I'd like to talk about the driving forces behind procrastination, such as 'worry' and 'fear.' …Additionally, I've included a link below that provides information on meditation/breathing techniques and stress relief methods recommended by experts.
Session 5	Primary conversation for a day: Reflecting on personal change<br>➢ Asking how to do for the rest of one's long-term goal that user had set<br>➢ Looking back for one's change after 4 sessions with MOA	MOA : Comparing before and now, do you see any positive changes since we started?<br>User : I've been managing my daily routine systematically, and I've reduced instances of missing or forgetting things.
