# Supplementary material for: Development of a Mobile Intervention for Procrastination Augmented With a Semigenerative Chatbot for University Students: Pilot Randomized Controlled Trial
Source: JMIR Mhealth Uhealth. 2025 Apr 10;13:e53133. doi: 10.2196/53133 (PMC12022524; doi:10.2196/53133)
Supplement: Multimedia Appendix 2 [file mhealth_v13i1e53133_app2.pdf]

Six Factors of Procrastination – Result of Literature Review

Physical and emotional stress

According to a meta-analysis of factors related to procrastination, procrastination is positively correlated with neuroticism caused by low self-esteem and self-efficacy, as well as depression and anxiety [1]. Individuals with low self-esteem and self-efficacy may experience a fear of failure, which can lead to procrastination or poorer performance [2]. Depression and anxiety are also highly correlated with low self-efficacy, which weakens the ability to control situations and contributes to procrastination [3]. In our data, we noted references to neuroticism related to emotional stress, such as lethargy and a depressive mood. Comments also mentioned physical illness or stress. We considered physical fatigue to fall under the same category as emotional stress, as physical health is highly correlated with mental well-being. Various solutions exist to address physical and emotional stress, depending on context and severity, but it is recommended to initially focus on recovery to prevent further performance decline [1].

Social relationship

Social relationships are an important factor in facilitating or inhibiting procrastination[4]. While they are related to emotional stress, we categorized them separately because the influence of peers or friends can act as distractions to performance [5]. Social support can enhance concentration on tasks and help reduce procrastination [6]. However, individuals often find it difficult to control their social relationships, making it necessary to redirect attention away from these issues or seek alternative social support.

Environmental distractors

The physical environment, including mobile phones and social media, is a significant factor contributing to distraction and temptation during performance [5]. Extensive internet use can lead students with low self-control to deviate from their goals and delay their work [7]. This can result in decreased concentration, ultimately lowering performance. To mitigate this factor, reducing distractions that capture one's interest has been shown to help students overcome procrastination [8]. Our data included leisure-related distractions that encourage procrastination, such as the temptation to hang out with friends or indulge in delicious food. Environmental distractors encompass both immaterial distractions and physical environments.

Perfectionism

Perfectionism can enhance conscientiousness in achieving one's goals but can also lead to procrastination, particularly in cases of socially prescribed perfectionism, which is linked to maladaptive perfectionism [9]. Socially prescribed perfectionism is driven by external expectations, where individuals strive to meet social criteria [10]. This can lead to maladaptive perfectionism characterized by worry rather than self-esteem, which is a risk factor for anxiety and depression [11]. Our data reflects procrastination factors related to perfectionism, such as "Struggling to meet expectations" and "Compulsion to be perfect on the first attempt." In cognitive behavioral therapy (CBT) addressing procrastination, it is recommended to first eliminate the fear of not meeting expectations [12]. Following this, stress-relieving strategies are needed, as students can easily experience stress due to high perfectionism [11].

Lack of motivation

Lack of motivation is a significant factor in procrastination, diminishing self-regulation abilities and leading to low performance [11]. It is associated with conscientiousness, where appropriate motivation to achieve goals fosters interest and concentration on tasks, thereby enhancing self-regulation for performance [13]. According to Temporal Motivation Theory, motivation is proportional to life values and expectations, highlighting the importance of establishing motivation for tasks by considering personal interests and values [14].

Low self-regulation

Procrastination is a failure of self-regulation influenced by a variety of factors from different perspectives. Self-regulation relates to conscientiousness and can be influenced by behavioral traits such as distractibility, goal-setting abilities, and achievement motivation [11]. A lack of concentration or feelings of weariness can exacerbate distractibility [15]. Establishing appropriate goals and planning one's career are effective strategies to address this factor. In this context, self-regulation is closely linked to environmental distractors and a lack of motivation.

1. Steel, P., *The nature of procrastination: a meta-analytic and theoretical review of quintessential self-regulatory failure.* Psychological bulletin, 2007. **133**(1): p. 65.
2. Judge, T.A. and J.E. Bono, *Relationship of core self-evaluations traits—self-esteem, generalized self-efficacy, locus of control, and emotional stability—with job satisfaction and*

job performance: A meta-analysis. Journal of applied Psychology, 2001. **86**(1): p. 80.

3. Lee, D.-g., K.R. Kelly, and J.K. Edwards, *A closer look at the relationships among trait procrastination, neuroticism, and conscientiousness.* Personality and Individual Differences, 2006. **40**(1): p. 27-37.
4. Nordby, K., K.B. Klingsieck, and F. Svartdal, *Do procrastination-friendly environments make students delay unnecessarily?* Social Psychology of Education, 2017. **20**(3): p. 491-512.
5. Svartdal, F., et al., *How study environments foster academic procrastination: Overview and recommendations.* Frontiers in Psychology, 2020. **11**: p. 3005.
6. Harris, N.N. and R.I. Sutton, *Task procrastination in organizations: A framework for research.* Human Relations, 1983. **36**(11): p. 987-995.
7. Quan-Haase, A. and A.L. Young, *Uses and gratifications of social media: A comparison of Facebook and instant messaging.* Bulletin of science, technology & society, 2010. **30**(5): p. 350-361.
8. Hinsch, C. and K.M. Sheldon, *The impact of frequent social Internet consumption: Increased procrastination and lower life satisfaction.* Journal of Consumer Behaviour, 2013. **12**(6): p. 496-505.
9. 김현이 and 송미경, *대학생의 사회부과 완벽주의가 학업지연행동에 미치는 영향: 정서조절양식의 매개효과.* 정서· 행동장애연구, 2018. **34**(4): p. 316-336.
10. Flett, G.L. and P.L. Hewitt, *Perfectionism: Theory, research, and treatment*. 2002: American Psychological Association.
11. Rice, K.G., C.M. Richardson, and D. Clark, *Perfectionism, procrastination, and psychological distress.* Journal of counseling psychology, 2012. **59**(2): p. 288.
12. Kuhl, J., *A functional-design approach to motivation and self-regulation: The dynamics of personality systems interactions*, in *Handbook of self-regulation*. 2000, Elsevier. p. 111-169.
13. Schunk, D.H., *Self-efficacy, motivation, and performance.* Journal of applied sport psychology, 1995. **7(2)**: p. 112-137.
14. Steel, P., Svartdal, F., Thundiyil, T., & Brothen, T., *Examining procrastination across multiple goal stages: a longitudinal study of temporal motivation theory.* Frontiers in psychology, 2018. **9**: p. 327.
15. Kuhl, J., *A functional-design approach to motivation and self-regulation: The dynamics of personality systems interactions*, in *In Handbook of self-regulation*. 2000. p. pp. 111-169.
