# Supplementary material for: Development of a Mobile Intervention for Procrastination Augmented With a Semigenerative Chatbot for University Students: Pilot Randomized Controlled Trial
Source: JMIR Mhealth Uhealth. 2025 Apr 10;13:e53133. doi: 10.2196/53133 (PMC12022524; doi:10.2196/53133)
Supplement: Multimedia Appendix 3 [file mhealth_v13i1e53133_app3.pdf]

Appendix 4.

	T0		T1		T2	
	Mean (SD)	95% CI	Mean (SD)	95% CI	Mean (SD)	95% CI
**PPS**						
Treatment	40.90 (7.32)	38.59 to 43.21	30.61 (8.00)	28.08 to 33.13	32.93 (9.39)	29.96 to 35.89
Control	38.26 (7.76)	35.56 to 40.97	30.59 (7.99)	27.8 to 33.37	30.76 (7.96)	27.99 to 33.54
**IPS**						
Treatment	31.93 (4.93)	30.37 to 33.48	27.39 (3.04)	26.43 to 28.35	25.73 (6.01)	23.83 to 27.63
Control	30.18 (4.96)	28.45 to 31.91	27.65 (2.66)	26.72 to 28.58	26.47 (5.05)	24.71 to 28.23
**TMBS**						
Treatment	45.2 (10.35)	41.93 to 48.46	52.76 (8.43)	50.09 to 55.42	53.95 (12.6)	49.97 to 57.93
Control	46.5 (12.09)	42.28 to 50.72	50.59 (9.58)	47.25 to 53.93	52 (13.68)	47.23 to 56.77
**Goal-setting/planning**						
Treatment	21.73 (4.41)	20.34 to 23.12	22.22 (3.75)	21.04 to 23.4	24.71 (4.59)	23.26 to 26.16
Control	21.91 (4.84)	20.22 to 23.60	21.24 (4.93)	19.52 to 22.96	23.59 (4.85)	21.9 to 25.28
**Procrastination**						
Treatment	10.10 (4.55)	8.66 to 11.53	14.71 (4.93)	13.15 to 16.26	14.02 (6.26)	12.05 to 16.00
Control	12.18 (5.58)	10.23 to 14.12	14.41 (4.67)	12.78 to 16.04	14.76 (5.48)	12.85 to 16.68
**Practice strategies**						
Treatment	13.37 (4.84)	11.84 to 14.89	15.83 (4.20)	14.50 to 17.16	15.22 (4.90)	13.67 to 16.77
Control	12.41 (5.41)	10.52 to 14.30	14.94 (4.18)	13.48 to 16.40	13.65 (5.56)	11.71 to 15.59
**ASRS**						
Treatment	36.10 (7.42)	33.76 to 38.44	35.98 (6.66)	33.87 to 38.08	39.10 (7.65)	36.68 to 41.51
Control	34.62 (8.25)	31.74 to 37.50	34.41 (7.12)	31.93 to 36.90	38.62 (7.54)	35.99 to 41.25
**PPS**						
Treatment	17.71 (5.63)	15.93 to 19.48	15.24 (2.82)	14.35 to 16.13	14.12 (6.44)	12.09 to 16.16
Control	17.38 (3.71)	16.09 to 18.68	16.00 (2.32)	15.19 to 16.81	15.35 (5.19)	13.54 to 17.16

Table S1 A summary of psychological and behavioral assessment for completers only

Engagement feature (sub-score)	Definition	Formula	Before adjusted		After adjusted	
			min-max	mean (SD)	range (min-max)	mean (SD)
App engagment						
Frequency of app access	The number of accessing the app	1-1/(*number of access* + 1)	0-11.71	2.76 (1.51)	0-1 (0-0.88)	0.63 (0.16)
Number of to-do lists	The number of to-do list sets per day	1-1/(*number of to do* + 1)	0-8.29	2.86 (1.49)	0-1 (0-0.88)	0.62 (0.17)
Elapsed time of the to-do list	The time difference (*diff_t)* between creation and completion of to-do	If *diff_t* <1hr, 0<br>elseif 1hr <=*diff_t* <12hr ,0.25<br>elseif 12hr <=*diff_t* <1day ,0.5<br>elseif 1day <= *diff_t* <7 day ,0.75<br>elseif *diff_t* > 7day, 1	0-7.77	0.44 (0.65)	0-1 (0-0.85)	0.25 (0.15)
Chatbot engagment						
Length of response	The length of users' response (*len_resp*) with the Moa	If *len_resp* < 3, 0<br>elseif 3 <= *len_resp* <4, 0.1<br>elseif 4 <= *len_resp* <6, 0.2<br>elseif 6 <= *len_resp* <9, 0.3<br>elseif 9 <= *len_resp* <13, 0.4<br>elseif 13 <= *len_resp* <18, 0.5<br>elseif 18 <= *len_resp* <26, 0.6<br>elseif 26 <= *len_resp* <38, 0.7<br>elseif 38 <= *len_resp* <55, 0.8<br>elseif 55 <= *len_resp* <80, 0.9<br>elseif *len_resp* >= 80, 1	1-426	13.62 (19.49)	0-1	0.15 (0.20)
Duration of response	The duration of users' response (*dur_resp*) with the Moa	If *dur_resp* <5s and *dur_resp* >= 132s, 0<br>elseif 5s <= *dur_resp* <6s, 0.125<br>elseif 6s <= *dur_resp* <8s, 0.25<br>elseif 8s <= *dur_resp* <12s, 0.375<br>elseif 12s <= *dur_resp* <20s, 0.5<br>elseif 20s <= *dur_resp* <36s, 0.625<br>elseif 36s <= *dur_resp* <68s, 0.75<br>elseif 68s <= *dur_resp* <132s, 1	0-140572	377.34 (4437.18)	0-1	0.25 (0.23)
Emotion score	The emotion of users' response with the Moa (exclude simple response)	extracted from KoBERT sentiment classifier (91.62% test accuracy)	-	-	0-1 (0.07-0.73)	0.32 (0.14)

Table S2 Specific engagement features (daily basis)

	T1-T0, mean (SD)		*F* value (*P* value)			
	Treatment (n=41)	Control (n=34)	T0 score	Time	Group	Interaction
**PPS**	-10.29 (7.74)	-7.68 (6.93)	207.36 [a]	124.61 [a]	2.63 (0.109)	2.55 (0.115)
**IPS**	-4.54 (5.59)	-2.53 (6.13)	78.81 [a]	46.28 [a]	1.95 (0.167)	3.51 (0.065)
**TMBS**	4.09 (9.69)	4.09 (9.69)	218.14 [a]	32.59 [a]	0.17 (0.683)	2.72 (0.104)
Goal-setting/planning	0.49 (4.71)	-0.68 (4.62)	171.22 [a]	0.01 (0.936)	0.65 (0.422)	1.37 (0.246)
Procrastination	4.61 (4.47)	2.24 (4.06)	287.18 [a]	57.01[a]	3.6 (0.062)	6.38 (0.014)
Practice strategies	2.46 (4.59)	2.53 (4.39)	243.24 [a]	28.41[a]	3.84 (0.054)	0 (0.944)
**ASRS**	-0.12 (6.12)	-0.21 (6.24)	335.53 [a]	0.06 (0.81)	5.24 (0.025)	0 (0.95)
**PSS**	-2.46 (4.64)	-1.38 (4.63)	176.26 [a]	21.17[a]	0.25 (0.618)	1.57 (0.214)

Table S3 Difference between T0, T1 with F statistic by linear mixed model setting scores at T0 as a covariate with completers

[a] The F value is significant at a significance level of <.001

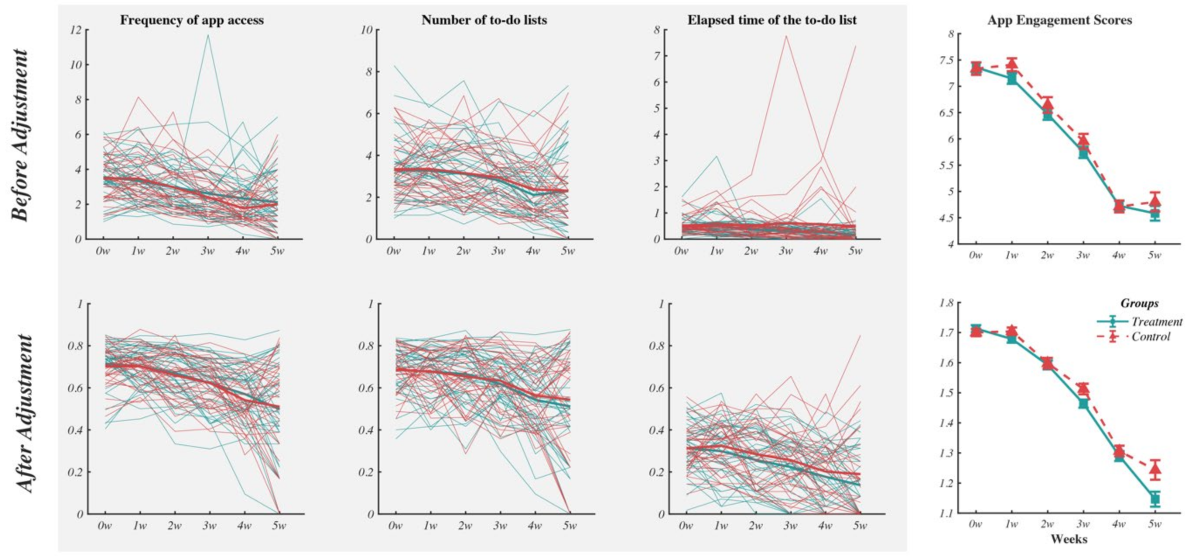

Figure S1 App engagement temporal pattern before and after adjustment (sub-score & overall)

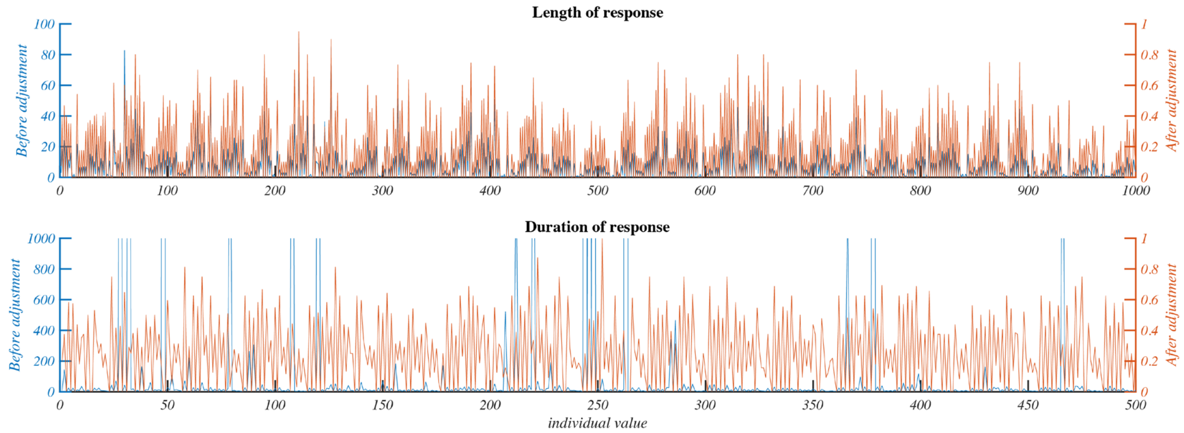

Figure S2 Chatbot engagement score before and after adjustment

Group difference (*P* value)	0w	1w	2w	3w	4w	5w
Adjusted						
Overall	.915	.357	.325	.434	.529	.432
Frequency of app access	.175	.566	.513	.750	.152	.886
Number of to-do lists	.621	.516	.741	.606	1.000	.689
Elapsed time of the to-do list	.915	.357	.325	.434	.529	.432
Before adjusted						
Overall	.721	.527	.300	.721	.584	.651
Frequency of app access	.717	.966	.455	.873	.060	.733
Number of to-do lists	.970	.919	.898	.713	.721	.802
Elapsed time of the to-do list	.706	.527	.300	.714	.584	.674

Table S4 Group differences in app engagement scores (two-sided Wilcoxon signed-rank test)

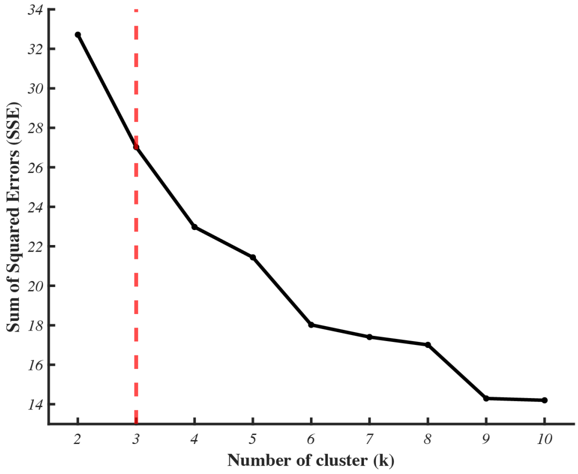

Figure S3 Optimal number of clusters

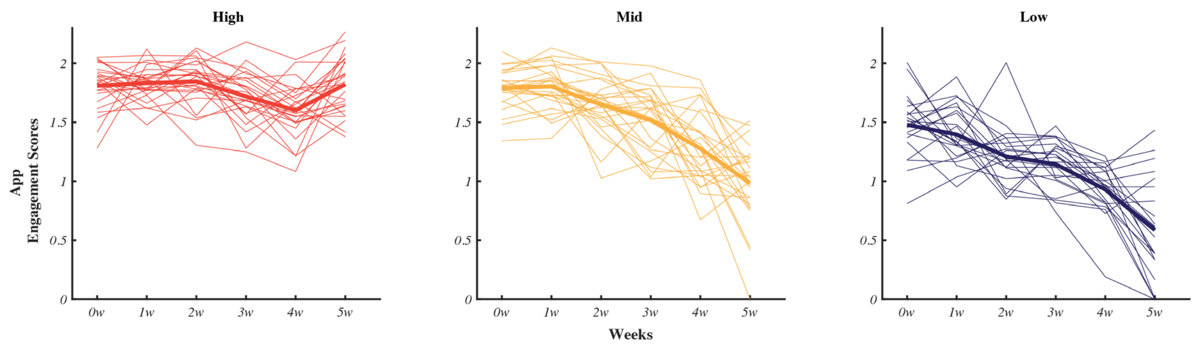

Figure S4 Temporal changes in app engagement scores within each cluster

	0w		1w		2w		3w		4w		5w	
	mean (SD)	95% CI	mean (SD)	95% CI	mean (SD)	95% CI	mean (SD)	95% CI	mean (SD)	95% CI	mean (SD)	95% CI
High	1.84 (0.18)	1.83 to 1.85	1.85 (0.15)	1.85 to 1.86	1.87 (0.16)	1.86 to 1.87	1.74 (0.19)	1.73 to 1.75	1.64 (0.20)	1.63 to 1.65	1.72 (0.34)	1.70 to 1.74
Mid	1.71 (0.16)	1.71 to 1.73	1.69 (0.22)	1.68 to 1.70	1.49 (0.29)	1.47 to 1.51	1.43 (0.26)	1.41 to 1.45	1.17 (0.23)	1.15 to 1.18	1.08 (0.34)	1.06 to 1.10
Low	1.46 (0.29)	1.43 to 1.49	1.43 (0.29)	1.40 to 1.46	1.29 (0.30)	1.26 to 1.32	1.12 (0.22)	1.10 to 1.14	0.90 (0.23)	0.88 to 0.92	0.39 (0.32)	0.36 to 0.42

Table S5 A summary of app engagement over time for each cluster

**Cluster difference (adjusted *P* value)**	**0w**	**1w**	**2w**	**3w**	**4w**	**5w**
High-Mid	.002	.002	.000	.000	.000	.000
Mid-Low	.001	.002	.015	.000	.000	.000

Table S6 The result of app engagement score difference by cluster (two-sided Wilcoxon signed-rank test)

	**High**		**Mid**		**Low**	
	T1-T0, mean (95% CI)	***Z*** (***P*** value)	T1-T0, mean (95% CI)	***Z*** (***P*** value)	T1-T0, mean (95% CI)	***Z*** (***P*** value)
**PPS**						
Treatment	-10.65 (-11.31 to -9.98)	3.41 (<.001)	-9.94 (-10.45 to -8.32)	3.08 (.002)	-10.82 (-12.45 to -9.18)	2.63 (.009)
Control	-8.00 (-8.78 to -7.22)	3.06 (.002)	-7.5 (-8.44 to -6.56)	2.64 (.008)	-7.43 (-9.92 to -4.94)	1.69 (.090)
**IPS**						
Treatment	-4.00 (-4.49 to -3.51)	2.67 (.008)	-5.08 (-5.83 to -4.32)	2.45 (.014)	-4.73 (-5.90 to -3.56)	1.91 (.056)
Control	-3.00 (-3.99 to -2.01)	1.33 (.183)	-0.79 (-1.44 to -0.13)	0.63 (.527)	-5.14 (-6.64 to -3.64)	2.00 (.046)
**TMBS**						
Treatment	5.94 (5.08 to 6.80)	-2.51 (.012)	7.85 (6.32 to 9.38)	-2.13 (.033)	9.73 (7.52 to 11.93)	-1.74 (.083)
Control	8.92 (7.44 to 10.40)	-2.38 (.017)	1.86 (0.87 to 2.84)	-1.10 (.271)	-0.43 (-2.68 to 1.83)	-0.32 (.752)
**Goal-setting/planning**						

Treatment	-0.06 (-0.54 to 0.42)	-0.16 (.876)	0.23 (-0.30 to 0.77)	0.21 (.833)	1.64 (0.69 to 2.58)	-0.67 (.504)
Control	1.85 (1.22 to 2.47)	-1.43 (.154)	-2.07 (-2.65 to -1.49)	1.46 (.145)	-2.57 (-3.32 to -1.82)	2.00 (.045)
**Procrastination**						
Treatment	4.94 (4.56 to 5.32)	-3.40 (<.001)	4.85 (4.19 to 5.50)	-2.91 (.004)	3.82 (2.92 to 4.72)	-2.04 (.041)
Control	3.46 (2.95 to 3.97)	-2.46 (.014)	2.07 (1.61 to 2.54)	-1.76 (.079)	0.29 (-1.13 to 1.70)	-0.68 (.498)
**Practice strategies**						
Treatment	1.06 (0.66 to 1.46)	-0.88 (.377)	2.77 (2.09 to 3.44)	-1.74 (.082)	4.27 (3.48 to 5.06)	-2.32 (.021)
Control	3.62 (2.95 to 4.28)	-2.46 (.014)	1.86 (1.44 to 2.27)	-2.01 (.045)	1.86 (0.30 to 0.09)	-0.68 (.497)
**ASRS**						
Treatment	-1.29 (-1.88 to -0.71)	0.59 (.553)	1.38 (-0.64 to 1.41)	-0.04 (.969)	1.09 (0.25 to 1.94)	-0.72 (.474)
Control	2.08 (1.22 to 2.94)	-0.90 (.366)	-1.64 (-2.42 to -0.87)	0.98 (.327)	-1.57 (-3.24 to 1.44)	0.68 (.499)
**PSS**						
Treatment	-2.71 (-3.12 to -2.29)	2.29 (.022)	-3.15 (-3.80 to -2.50)	2.17 (.030)	-1.27 (-2.18 to -0.37)	0.76 (.449)
Control	-1.69 (-2.26 to -1.13)	1.43 (.154)	-0.93 (-1.56 to -0.29)	0.76 (.449)	-1.71 (-3.18 to -0.25)	0.76 (.446)

Table S7 Difference between T0, T1 within group in each cluster via Wilcoxon signed-test (not corrected version for multiple testing)

Dimension (min – max)	Mean (SD)		Percentage >normal (%)		
	Treatment	Control	Treatment	Control	p-value
Usability (25-100)	72.8(16.0)	75.15(10.13)	71.0	73.8	0.386
Acceptability (8 - 48)	34.73(7.35)	33.97(6.97)	80.8	73.2	0.026
Feasibility (6 – 36)	24.76(3.49)	25.41(3.72)	73.6	74.5	0.822
Satisfaction (8 – 32)	23.51(4.22)	23.35(4.73)	77.1	72.8	0.221

Table S8 The result of the user experience surveys with mean and standard deviation (SD), indicating that over 70% of responses exceeded the 'normal' in all surveys.
